# Prehabilitation in Patients Undergoing Cardiac Procedures: A Systematic Review and Meta-Analysis

**DOI:** 10.1016/j.jacadv.2026.102587

**Published:** 2026-01-29

**Authors:** Carolin Steinmetz, Phuc Thien Tran, Stephanie Heinemann, Daniel Arroyo-Ariza, Jane Jurayj, Nicole B. Katz, Johanneke Hartog, Thomas Schmidt, Bart Scheenstra, Hermioni L. Amonoo, Elizabeth N. Madva, Jason Z. Qu, Oluwaseun Akeju, Jeffery C. Huffman, Ingo Kutschka, Christoph Herrmann-Lingen, Julie K. Silver, Christine A.F. von Arnim, Anna Lee, Christian Röver, Christopher M. Celano, Monika Sadlonova, Cardiac Prehabilitation Network

**Affiliations:** a Department of Geriatrics, University Medical Center Göttingen, Göttingen, Germany; b Department of Public Health, Health Services Research and Health Technology Assessment, Institute of Public Health, Medical Decision Making and Health Technology Assessment, UMIT TIROL - University for Health Sciences and Technology, Hall in Tirol, Austria; c Department of Medical Statistics, University Medical Center Göttingen, Göttingen, Germany; d Barnes Jewish Hospital, Washington University in St. Louis, St. Louis, USA; e University of Rochester School of Medicine and Dentistry, Rochester, New York, USA; f Department of Physical Medicine and Rehabilitation, Spaulding Rehabilitation Hospital, Charlestown, Massachusetts, USA; g Department of Physical Medicine and Rehabilitation, Harvard Medical School, Boston, Massachusetts, USA; h Department of Cardio-Thoracic Surgery, University of Groningen, University Medical Center Groningen, Groningen, Groningen, The Netherlands; i Schüchtermann-Schiller'sche Clinic, Bad Rothenfelde, Germany; j Institute of Cardiology and Sports Medicine, Department Preventive and Rehabilitative Sport and Exercise Medicine, German Sport University, Cologne, Germany; k Department of Cardiac Rehabilitation, Leiden, The Netherlands; l Department of Psychiatry, Brigham and Women's Hospital, Boston, Massachusetts, USA; m Department of Psychiatry, Harvard Medical School, Boston, Massachusetts, USA; n Department of Supportive Oncology, Dana-Farber Cancer Institute, Boston, Massachusetts, USA; o Department of Psychiatry, Massachusetts General Hospital, Boston, Massachusetts, USA; p Department of Anesthesiology, Mass General Brigham, Massachusetts General Hospital, Harvard Medical School, Boston, Massachusetts, USA; q Department of Cardiovascular and Thoracic Surgery, University Medical Center Göttingen, Göttingen, Germany; r German Center for Cardiovascular Research (DZHK), Partner Site Lower Saxony, Göttingen, Germany; s Department of Psychosomatic Medicine and Psychotherapy, University Medical Center Göttingen, Göttingen, Germany; t Department of Orthopaedic Surgery and Rehabilitation, Wake Forest University School of Medicine, Winston-Salem, North Carolina, USA; u Department of Anaesthesia and Intensive Care, Chinese University of Hong Kong, Shatin, Hong Kong SAR, China

**Keywords:** cardiac procedure, cardiac surgery, meta-analysis, prehabilitation, preoperative preparation, systematic review

## Abstract

**Background:**

Evidence supporting prehabilitation before cardiac procedures is growing, but the efficacy of different components remains unclear.

**Objectives:**

The primary aim was to assess the efficacy of prehabilitation on clinical outcomes based on recent randomized controlled trials (RCTs). The secondary aim was to identify effective intervention and which patient subgroups benefit most.

**Methods:**

We searched Medline, Web of Science, PsycINFO, Embase, Scopus, and Cochrane Central Register of Controlled Trials Library for RCTs comparing prehabilitation with standard care in cardiac patients up to August 2024. Trials were screened by 2 reviewers and meta-analyses were performed using random-effects models.

**Results:**

Forty-four RCTs including 3,925 patients were identified. Prehabilitation improved preprocedural functional capacity (6-minute walk distance) and recovery (in-hospital length of stay, intensive care unit stay, and occurrence of postprocedural pneumonia). Six trials (n = 600) showed improved 6-minute walk distance (mean difference [MD] 68.87 m; 95% CI: 12.76-124.98 m; *P* = 0.020). In 18 studies (n = 1,568), length of stay was shorter (MD -0.95 days; 95% CI: −1.77 to −0.13 days; *P* = 0.026) and meta-regression showed greater effect in studies including more women (*P* = 0.015). In 16 trials (n = 1,149), intensive care unit stay was reduced (MD −6.03 hours; 95% CI: −12.01 to −0.06 hours; *P* = 0.048). In 5 studies (n = 729), postprocedural pneumonia occurred less frequently (OR: 0.33; 95% CI: 0.15-0.72; *P* = 0.017). The analysis revealed substantial heterogeneity and risk of bias. Analysis of specific components showed no consistent effects.

**Conclusions:**

Prehabilitation before cardiac procedures may enhance preprocedural functional capacity and postprocedural recovery, particularly in women. Further multicenter studies are needed.

Cardiac surgery carries a high risk of developing postoperative complications, including postoperative atrial fibrillation (7%-53%),^1^ atelectasis (30% to 72%), pleural effusion (24%-63%), and pneumonia (2%-20%),^2^ which significantly impact in-hospital length of stay (LOS), quality of life,^3^ and physical functioning. Both sociodemographic and medical factors, including older age, frailty, sarcopenia, reduced exercise capacity, and loss of physical functioning, increase the risk of complications following cardiac surgery.^4^^,^^5^ Female sex is an additional and often underestimated risk factor for postcardiac surgery complications.^6^^,^^7^

Enhanced Recovery After Surgery (ERAS) protocols for perioperative care in cardiac surgery aim to improve postoperative outcomes by addressing these pre-existing risk factors.^8^

Prehabilitation is a key preoperative ERAS strategy that is conducted weeks ahead of an elective cardiac procedure; it can include a single intervention (unimodal) or multiple interventions (multimodal)^8^^,^^9^ and involves optimizing patients’ physical and mental status as well as maximizing functional reserve in preparation for elective surgery and subsequent recovery.^10^ Currently, there is no universally accepted definition of prehabilitation recognized by professional associations.^11^

Since 2000, cardiac prehabilitation research has rapidly expanded.^12^ Previous published systematic reviews and meta-analyses have reported that cardiac prehabilitation can shorten LOS^13^, ^14^, ^15^, ^16^, ^17^ and intensive care unit (ICU) stay,^18^ improve functional capacity,^13^^,^^18^ and reduce postoperative complications, such as postoperative atrial fibrillation^13^^,^^16^^,^^17^ and pulmonary complications.^15^^,^^19^ However, high heterogeneity and a small number of included studies have limited the validity and generalizability of their findings.^13^, ^14^, ^15^, ^16^^,^^18^

The Joint Consensus Statement on Perioperative Care in Cardiac Surgery from the ERAS Cardiac Society, ERAS International Society, and The Society of Thoracic Surgeons emphasizes the importance of evaluating both the efficacy and the core components of prehabilitation programs.^20^ Therefore, the primary aim of this systematic review and meta-analysis was to update prior reviews and assess the efficacy of prehabilitation on clinical outcomes based on a larger number of recent randomized controlled trials (RCTs). The secondary aim was to identify effective intervention components and determine which patient subgroups benefit the most (eg, by age, sex, or procedure type) from prehabilitation.

## Methods

### Protocol, registration, and study eligibility criteria

This systematic review and meta-analysis follows the Preferred Reporting Items of Systematic Reviews and Meta-Analyses guidelines^21^ (see Supplemental Table 6). The protocol has been published previously in the International Prospective Register of Systematic Reviews (PROSPERO) registry (CRD42022346710). Inclusion criteria are listed in Table 1. Excluded were case reports, case series, methods papers, studies without outcome data, intervention studies lacking a preoperative component, abstracts, and presentations.

**Table 1:** Inclusion Criteria for Literature Selection

Age	Adults ≥18 y old
Type of cardiac procedure	Patients before nonurgent cardiac procedure such as CABG (on-pump or off-pump), surgical valve replacement, or TAVR, but not percutaneous coronary intervention or electrophysiological interventions
Intervention	Inpatient or outpatient preoperative/preprocedural (“prehabilitation”) interventions before a cardiac procedure which included at least 1 of the following domains: aerobic or anaerobic conditioning, muscle training, respiratory muscle training, cardiovascular risk factor modification, nutrition, sleep hygiene, psychoeducation, psychological intervention, or cognitive training
Duration	≥1 wk before cardiac procedure
Control	Standard medical care without preoperative/preprocedural intervention before nonurgent cardiac procedure
Outcomes	Functional capacity (eg, 6-minute walk test), muscle strength (eg, hand grip strength), periprocedural and postprocedural complications (cardiac and non cardiac complications), frailty, cognitive outcomes (eg, objective and subjective memory impairment), psychological outcomes (eg, anxiety, depression, stress, expectations), sleep quality, of quality of life, recovery status (length intensive care unit and hospital stay), cardiac-related symptoms (eg, dyspnea or angina pectoris), in-hospital, and all-cause mortality
Accepted study designs	Randomized controlled trials; published in English

CABG = coronary artery bypass graft surgery; TAVR = transcatheter aortic valve replacement.

### Data sources, search strategies, and identification of studies

The search strategy was based on the PICOS framework (Population, Intervention, Control, Outcome, Study Design).^22^ Systematic searches of PubMed, Web of Science, PsycINFO, Embase, and Cochrane Central Register of Controlled Trials and Scopus were conducted from inception until June 30, 2022, using keywords related to cardiac prehabilitation. The search was updated for articles published until August 31, 2024. Full search details are available in Supplemental Table 1.

### Outcomes definition

The primary objective of this systematic review and meta-analysis was to assess the efficacy of prehabilitation on clinical outcomes. A secondary objective was to identify effective intervention components and the patient subgroups most likely to benefit (eg, by age, sex, or procedure type). Prospectively defined clinical outcome definitions registered in PROSPERO are provided in Supplemental Table 2.

### Study selection and data extraction

Identified articles were imported into Covidence. After removing duplicates, titles, abstracts, and full texts were screened twice by independent reviewers (M.S., C.S., S.H., D.A.A., J.J., N.B.K., J.H., B.S., T.S., and E.N.M.) based on PICOS criteria (Table 1). Disagreements were resolved by C.C. Data on study characteristics, intervention details, and outcomes were extracted into a preformatted Excel sheet by study team members (M.S., C.S., S.H., N.B.K., J.H., B.S., T.S., and E.N.M.). P.T.T. extracted numerical outcome values for meta-analysis in R. Data extraction was verified by M.S., C.S., and S.H. Evidence quality was assessed independently by C.S. and S.H. using Grading of Recommendations Assessment, Development, and Evaluation (GRADE) (GRADE Pro GDT), categorizing certainty as high, moderate, low, or very low.^22^^,^^23^

### Assessment of risk of bias domains

Risk of bias (RoB) in RCTs was assessed using the Cochrane Collaboration Risk of Bias tool 2.^24^ Two reviewers (C.S. and S.H.) independently rated RoB, with a third reviewer (M.S.) resolving disagreements. Authors were contacted for missing data. RoB visualization was generated with the *robvis* tool.^25^

### Statistical analysis and data synthesis

A meta-analysis was conducted for each of the following outcomes: 6-minute walk distance (6MWD) preprocedure and postprocedure, in-hospital LOS, ICU stay, in-hospital and follow-up all-cause mortality, anxiety postprocedure, quality of life preprocedure and postprocedure, hand grip strength (HGS) preprocedure, and cardiac and noncardiac complications postprocedure (for details see Supplemental Table 2 for a list of predefined complications). Treatment effects were quantified as mean differences (MDs) for 6MWD, in-hospital LOS, and ICU stay. All-cause mortality as well as cardiac and noncardiac postprocedural complications (atrial fibrillation, pneumonia, atelectasis pleural effusion, delirium, and infection) were analyzed based on the OR. As anxiety (State-Trait Anxiety Inventory Anxiety-Trait,^26^ State-Trait Anxiety Inventory Anxiety-State,^27^ and Hospital Anxiety and Depression Scale subscale anxiety^27^, ^28^, ^29^, ^30^) and quality of life (Short Form-36,^12^^,^^28^^,^^29^^,^^31^, ^32^, ^33^ Short Form-12,^26^^,^^30^^,^^34^ MacNew Heart Disease Health-related Quality of Life,^35^ and European Quality of Life 5 Dimensions Version^36^ questionnaire) were reported on different scales, standardized MD (SMD) was used for analyses.

In case a trial included more than 2 treatment arms, only the pairwise comparison deemed most relevant was considered. Medians and IQRs were converted to mean ± SD based on a normal approximation using the formula by Wan et al.^37^ Random-effects models, using the restricted maximum likelihood approach, were used for meta-analyses to account for potential heterogeneity between studies. CIs were derived using the Knapp-Hartung method with ad hoc adjustment.^38^ Forest plots illustrate meta-analysis results, including measures of heterogeneity (τ and I^2^). I^2^ expresses the proportion of variability in a meta-analysis, which is explained by between-study heterogeneity rather than by sampling error. I^2^ over 50% means between-study heterogeneity explains over 50% variability in the meta-analysis. RCTs with no events in either arm were excluded from forest plots.^22^ For outcomes with >10 studies, meta-regression assessed heterogeneity sources (age, sex, procedure type, study country, prehabilitation type, and duration of prehabilitation). Studies missing covariate data were omitted from specific analyses. Analyses were performed in R programming language, using the meta and metafor packages (version 4.3.3, R Foundation for Statistical Computing).

## Results

### Search process

**Figure 1:**
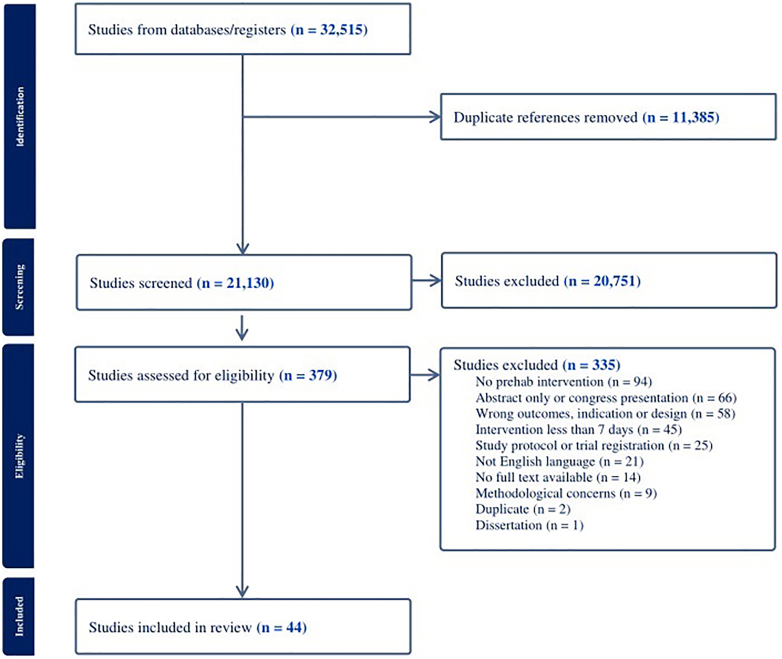
Flow Diagram of Study Inclusion and Exclusion Process

Database searches identified 32,515 titles and abstracts. After removing duplicates and studies that did not meet the inclusion criteria, 21,130 titles and abstracts were screened for eligibility. Of these articles, 20,751 were excluded, primarily because they were not thematically relevant or the study cohort was not suitable. The remaining 379 full texts were reviewed, and 335 were excluded. The reason for study exclusions are described in Figure 1. The most common reasons for exclusion were methodological concerns. A total of 44 studies were included in the systematic review and meta-analysis (Figure 1).

### Characteristics of the included studies

These 44 studies involved 3,925 patients, of whom 1,964 were in the intervention group and 1,961 in the control group. All studies were published between 1980 and 2024 in 17 different countries. Twenty-six studies are RCTs,^12^^,^^26^, ^27^, ^28^^,^^30^^,^^34^, ^35^, ^36^^,^^39^, ^40^, ^41^, ^42^, ^43^, ^44^, ^45^, ^46^, ^47^, ^48^, ^49^, ^50^, ^51^, ^52^, ^53^, ^54^, ^55^, ^56^ 8 are randomized placebo-controlled trials,^57^, ^58^, ^59^, ^60^, ^61^, ^62^, ^63^, ^64^ and 6 are RCTs with a pilot^32^^,^^65^, ^66^, ^67^ or feasibility^33^^,^^68^ approach. Three studies are secondary analyses of RCTs^29^^,^^31^^,^^69^ and 1 is a multicenter RCT.^70^ Furthermore, the RCTs from Auer et al.^40^, Rief et al.,^30^ and Salzmann et al^50^ all analyzed data from the PSY-HEART (Pre-surgery optimization of patient’s expectations to improve outcome in heart surgery) trial. Thirty-two trials^26^^,^^27^^,^^29^, ^30^, ^31^^,^^34^^,^^40^, ^41^, ^42^, ^43^^,^^45^^,^^48^, ^49^, ^50^, ^51^^,^^53^, ^54^, ^55^, ^56^, ^57^, ^58^, ^59^, ^60^, ^61^, ^62^, ^63^, ^64^, ^65^, ^66^, ^67^, ^68^^,^^70^ analyzed the effects of unimodal prehabilitation interventions, whereas twelve^12^^,^^28^^,^^32^^,^^33^^,^^35^^,^^36^^,^^39^^,^^44^^,^^46^^,^^47^^,^^52^^,^^69^ examined the benefit of a multimodal approach (>1 module). The content of prehabilitation intervention modules was diverse, for example, including exercise, cognitive training, breathing therapy, supplementation, relaxation, and psychological intervention. The range of the prehabilitation intervention duration was at least 1 week and up to 10 months before surgery. Subgroup analyses for all meta-analyses according to unimodal vs multimodal programs and duration (2 weeks vs <2 weeks) are available in Supplemental Figures 10, 11, and 12. In addition, all meta-analyses were performed without breathing-only studies and supplement/medication-only studies (see Supplemental Figures 13, 14, and 15. A detailed description of the control groups, prehabilitation interventions and modules carried out in the included studies can be found in Supplemental Table 3.

Seven studies reported data on safety.^12^^,^^32^^,^^35^^,^^36^^,^^44^^,^^66^^,^^69^ Exercise-based prehabilitation was classified as safe in five^12^^,^^32^^,^^35^^,^^44^^,^^69^ of the 6 studies assessing this outcome.^12^^,^^32^^,^^35^^,^^36^^,^^44^^,^^69^ In the study by Akowuah et al,^36^ the rate of adverse events during prehabilitation was higher than in the standard care group; however, only a minority of these events were related to the intervention. The authors suggested that this difference may be attributable to observer bias, as patients in the prehabilitation group were admitted to hospital more often and were more frequently asked about adverse events than those in the standard care group.^36^ Hulzebos et al^66^ additionally confirm the safety of an inspiratory muscle training before a coronary artery bypass graft surgery.

### Quality of the included studies

**Figure 2:**
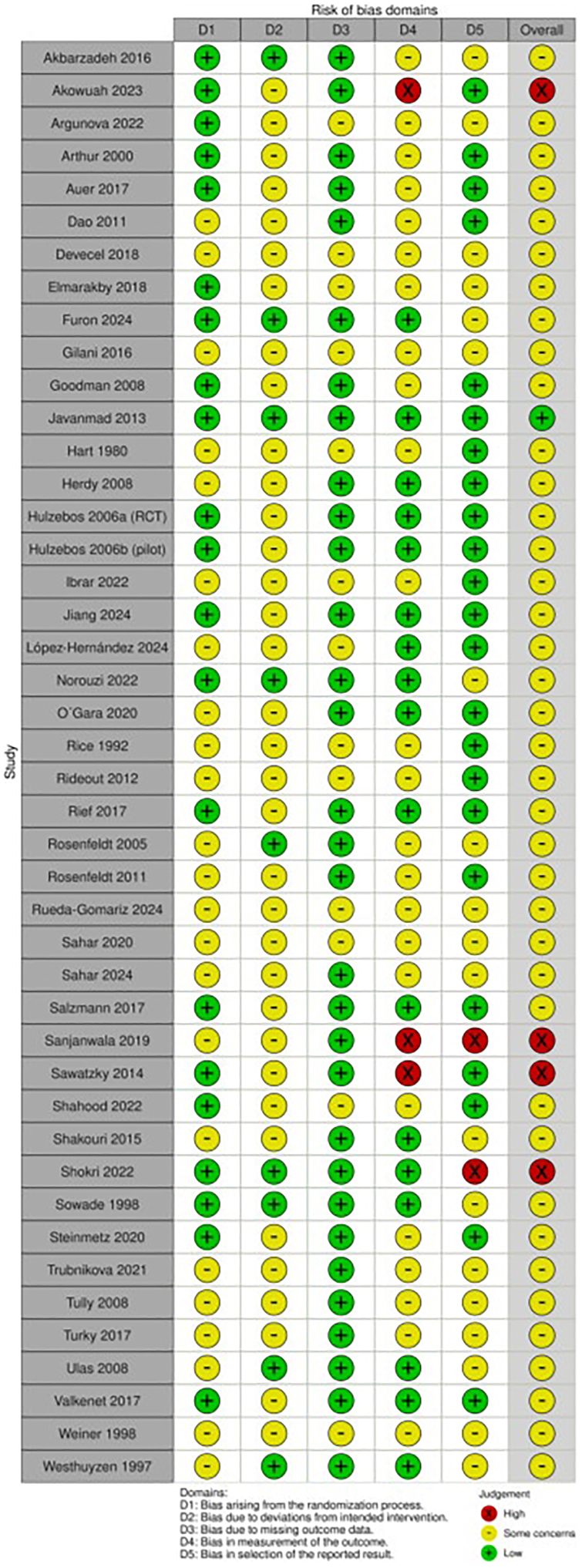
**Risk of Bias Assessments According to Risk of Bias 2 of the Included Studies** RCT = randomized controlled trial.

Detailed results of the RoB evaluation are listed in Figure 2. Inadequate reporting of allocation concealment (D1) was found in 23 studies.^26^^,^^27^^,^^29^^,^^33^^,^^34^^,^^41^^,^^43^^,^^44^^,^^46^, ^47^, ^48^, ^49^^,^^52^, ^53^, ^54^, ^55^, ^56^^,^^59^^,^^62^^,^^63^^,^^67^, ^68^, ^69^ Many studies (n = 35) showed some concerns in column D2 because the type of intervention did not allow for blinding of patients and clinical staff during the performance of the intervention.^12^^,^^26^, ^27^, ^28^, ^29^, ^30^, ^31^, ^32^, ^33^, ^34^, ^35^, ^36^^,^^39^, ^40^, ^41^^,^^43^, ^44^, ^45^, ^46^, ^47^, ^48^, ^49^, ^50^, ^51^, ^52^, ^53^, ^54^, ^55^, ^56^^,^^65^, ^66^, ^67^, ^68^, ^69^, ^70^ Thirteen studies had concerns about missing outcome data (D3), mainly because the number of outcomes analyzed was not described in the results section or a high number of dropouts were reported in 1 group.^27^^,^^29^^,^^39^^,^^41^^,^^43^^,^^46^, ^47^, ^48^^,^^51^^,^^55^^,^^56^^,^^65^^,^^69^ There were some concerns about the measurement of outcomes in 24 studies, as the blinding of the assessors was not described in detail.^12^^,^^26^, ^27^, ^28^, ^29^^,^^33^^,^^35^^,^^39^, ^40^, ^41^^,^^43^^,^^46^, ^47^, ^48^, ^49^^,^^51^^,^^53^, ^54^, ^55^, ^56^, ^57^^,^^59^^,^^65^^,^^67^ Nineteen studies were rated with some concerns about the selection of the reported results (D5), mainly because the trials were not prospectively registered, no study protocol existed, and/or primary and secondary outcomes were not specified in the article.^39^^,^^41^, ^42^, ^43^^,^^48^^,^^49^^,^^52^, ^53^, ^54^, ^55^, ^56^, ^57^^,^^59^^,^^61^, ^62^, ^63^, ^64^, ^65^^,^^67^

Four studies^32^^,^^34^^,^^36^^,^^60^ were rated in summary with a high risk because their limitations stated that the assessors involved were not blinded^32^^,^^34^^,^^36^ and/or the primary outcome(s) described in the prospective registry were not consistent with the primary outcome(s) reported in the article.^34^^,^^60^ The best quality is shown in the placebo-controlled trials.^58^^,^^60^^,^^61^^,^^64^ The overall quality of evidence is summarized for each outcome according to GRADE guidelines in Supplemental 1 (Supplemental Table 4).

### Primary outcomes

#### Recovery status

**Figure 3:**
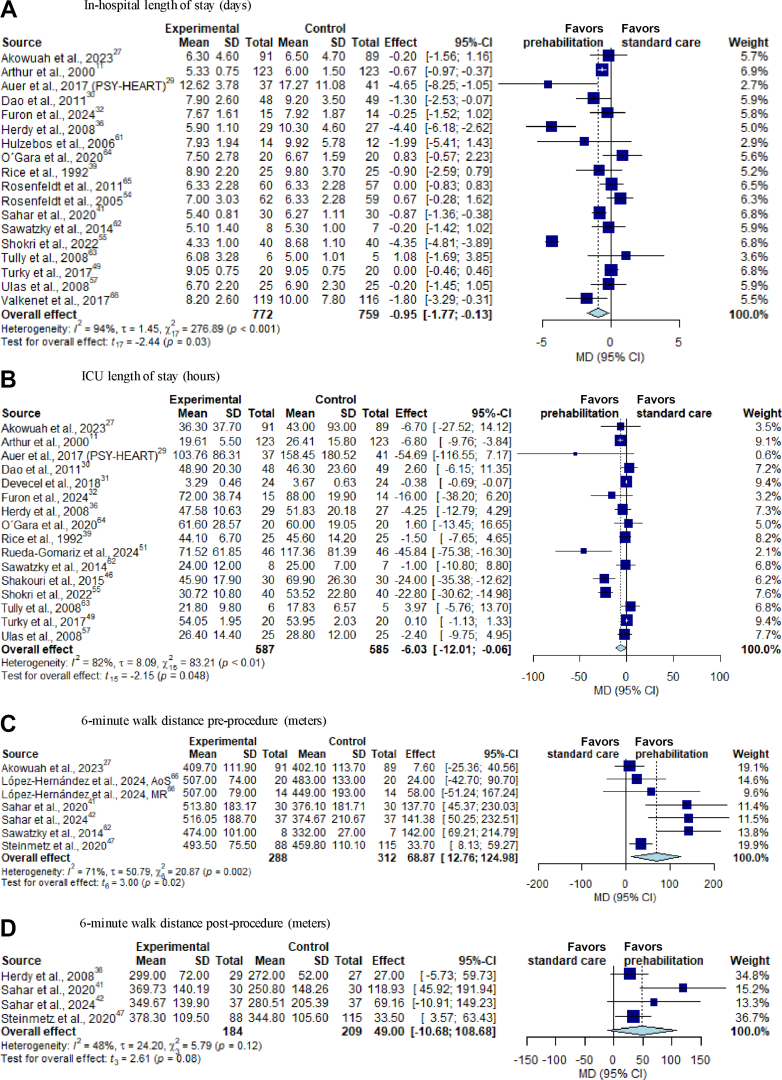
**Forest Plots of Recovery Status and Perioperative Functional Capacity** (A) In-hospital length of stay; (B) ICU length of stay; (C) 6-minute walk distance preprocedure; (D) 6-minute walk distance postprocedure. CI = confidence interval; ICU = intensive care unit; MD = mean difference; SD = standard deviation.

Eighteen studies including 1,531 patients reported the duration of in-hospital LOS.^12^^,^^26^^,^^31^, ^32^, ^33^^,^^36^^,^^40^^,^^42^^,^^44^^,^^45^^,^^47^^,^^48^^,^^54^^,^^59^^,^^60^^,^^62^^,^^67^^,^^68^ Participation in a prehabilitation intervention significantly decreased in-hospital LOS in comparison to controls (MD −0.95 days; 95% CI: −1.77 to −0.13 days; *P* = 0.026; I^2^ = 94%; GRADE low) (Figure 3A).

The duration of ICU stay was assessed in sixteen studies including 1,172 patients.^12^^,^^26^^,^^32^^,^^36^^,^^40^, ^41^, ^42^^,^^44^^,^^47^^,^^52^^,^^54^^,^^56^^,^^60^^,^^62^^,^^67^^,^^68^ Patients in the prehabilitation group spent significantly less time in the ICU than patients of the control group (MD −6.03 hours; 95% CI: −12.01 to −0.06 hours; *P* = 0.048; I^2^ = 82%; GRADE low) (Figure 3B).

#### Functional capacity, quality of life, and psychological outcome

The preprocedural 6MWD was analyzed in 6 trials including 600 patients.^32^^,^^35^^,^^36^^,^^48^^,^^49^^,^^69^ The data confirmed a significant effect in favor of the prehabilitation group (MD 68.87 m; 95% CI: 12.76-124.98 m; *P* = 0.020; GRADE low) (Figure 3C). The postprocedural 6MWD was reported before hospital discharge in 4 studies including 393 patients.^35^^,^^44^^,^^48^^,^^49^ The pooling of data did not show a significant effect in postprocedural 6MWD at hospital discharge in participants of the prehabilitation group compared to controls (MD 49.00 m; 95% CI: -10.68-108.68 m; *P* = 0.079; I^2^ = 48%; GRADE moderate) (Figure 3D). Likewise, there were no improvements in 6MWD with prehabilitation over controls up to 12 weeks after cardiac surgery (Supplemental Figures 1 and 2). The preprocedural HGS was investigated in 2 studies with 248 patients,^36^^,^^69^ with no significant effect in favor of prehabilitation intervention (MD 2.35 kg; 95% CI: −0.89 to 5.58 kg; *P* = 0.090; GRADE moderate) (Supplemental Figure 3).

Change in global quality of life between initiation of prehabilitation until immediately before the procedure was assessed in 2 studies including 383 patients using the MacNew questionnaire and the EUROQOL-5D showing no significant effect^35^^,^^36^ (SMD: 0.11; 95% CI: −1.18 to 1.39; *P* = 0.48; I^2^ = 0%; GRADE moderate) (Supplemental Figure 4). Quality of life was measured 4 to 6 weeks after the cardiac procedure and compared to the beginning of prehabilitation in 3 studies including 480 patients with 3 different assessments with no significant effect (EQ-5D-5 L, SF-12, MacNEW questionnaire)^26^^,^^35^^,^^36^ (SMD: −0.07; 95% CI: −0.50 to 0.36; *P* = 0.55; I^2^ = 16%; GRADE moderate) (Supplemental Figure 5 in Supplemental 1).

Postprocedural anxiety assessed by State-Trait Anxiety Inventory was reported in 2 studies including 137 patients.^26^^,^^27^ The change in anxiety did not differ significantly between patients participating in the prehabilitation group compared to control group immediately before hospital discharge (SMD: −1.10; 95% CI: −4.54-2.34; *P* = 0.15; I^2^ = 48%; GRADE moderate) (Supplemental Figure 6).

#### Postoperative complications

**Figure 4:**
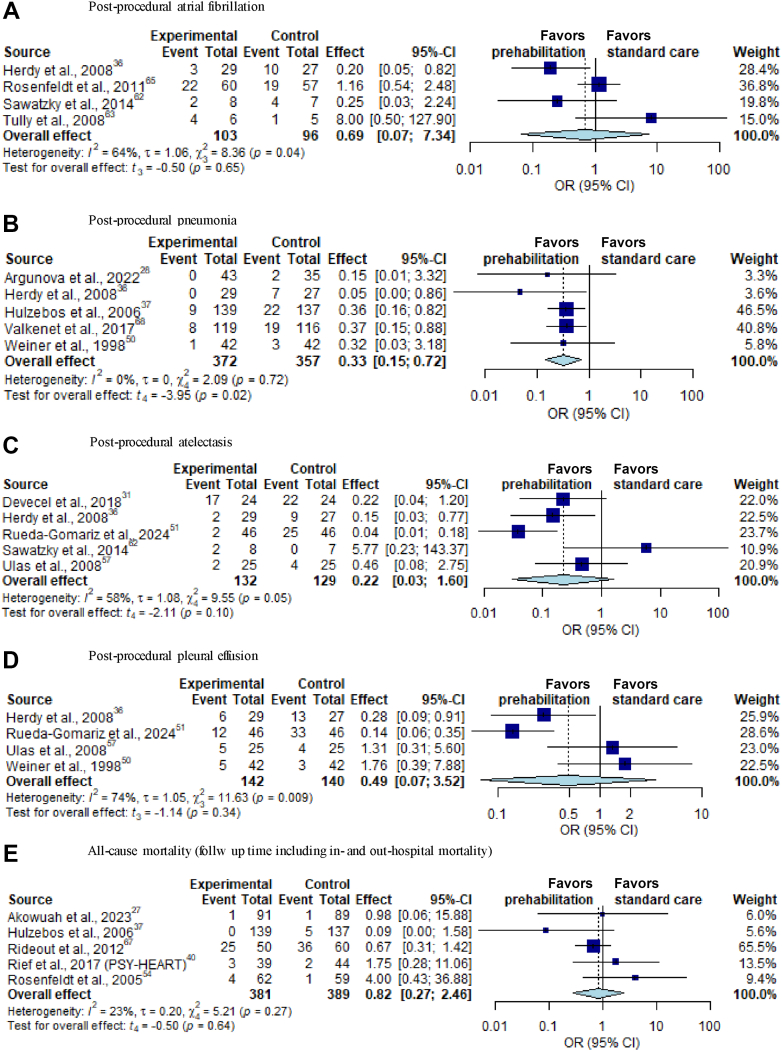
Forest Plots of Selected Postoperative Complications (A) Postprocedural atrial fibrillation; (B) postprocedural pneumonia; (C) postprocedural atelectasis; (D) postprocedural pleural effusion; (E) all-cause mortality. CI = confidence interval; OR = odds ratio; SD = standard deviation.

The occurrence of atrial fibrillation was assessed in 4 studies including 199 patients^32^^,^^33^^,^^44^^,^^67^; no significant overall effect of prehabilitation was observed (OR: 0.69; 95% CI: 0.07-7.34; *P* = 0.65; I^2^ = 64%; GRADE moderate) (Figure 4A). In 5 studies including 729 patients, a significant decrease in the occurrence of postprocedural pneumonia was observed in the prehabilitation group in comparison to controls (OR: 0.33; 95% CI: 0.15-0.72; *P* = 0.017; GRADE moderate; I^2^ = 0%) (Figure 4B).^31^^,^^39^^,^^44^^,^^45^^,^^55^ No significant effects were found in the pooled data of the studies for the occurrence of postprocedure atelectasis,^32^^,^^41^^,^^44^^,^^56^^,^^62^ pleural effusion,^44^^,^^55^^,^^56^^,^^62^ delirium^68^^,^^70^ and infection^60^^,^^62^ (Figures 4C and 4D, Supplemental Figures 7 and 8) (GRADE: low-high). Similarly, prehabilitation intervention did not significantly impact all-cause mortality^29^^,^^30^^,^^36^^,^^45^^,^^59^ (Figure 4E) (GRADE: moderate). Because of the lack of available data, other preprocedural and postprocedural clinical outcomes could not be included in this meta-analysis.

### Secondary and further outcomes

A meta-regression was performed for the variables in-hospital LOS and ICU stay, as ≥10 studies each reported on these outcomes. The meta-regression showed that the effect of prehabilitation on in-hospital LOS was significantly more beneficial in studies with a higher number of females (*P* = 0.015) (Supplemental Figure 9, Supplemental Table 5). No significant effects of the different prehabilitation components and further covariables were found (Supplemental Table 5). A summary of the most important findings can be found in the Central Illustration.

**Central Illustration:**
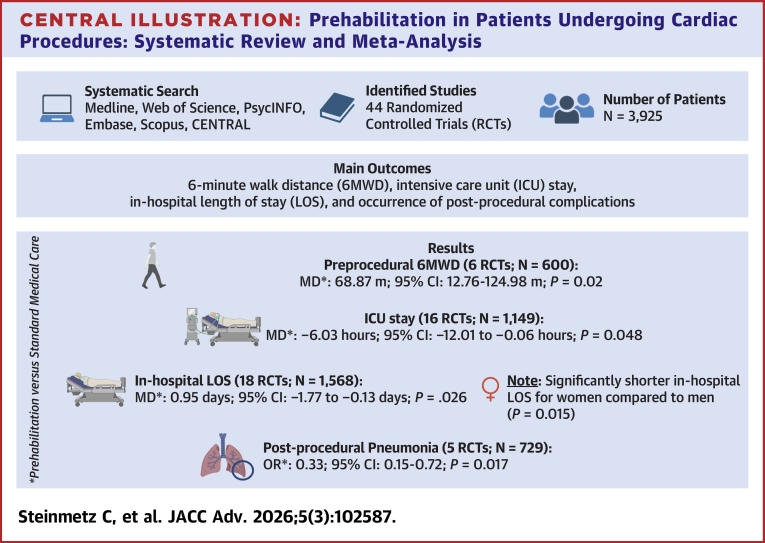
**Prehabilitation in Patients Undergoing Cardiac Procedures: Systematic Review and Meta-Analysis** ∗Prehabilitation versus standard medical care.

## Discussion

This is a comprehensive systematic review and meta-analysis including 44 RCTs on prehabilitation interventions performed ≥7 days before cardiac procedures. Our results indicate that prehabilitation significantly improved preprocedural functional status (6MWD) and postprocedural recovery (ie, LOS, ICU stay, and occurrence of postprocedural pneumonia). The prehabilitation group exhibited a decreased incidence of postoperative atelectasis, although the difference was not statistically significant.

Furthermore, meta-regression suggested greater efficacy of cardiac prehabilitation in women.

Prehabilitation reduced LOS by 1 day and ICU stay by 6 hours, which may be clinically relevant. Even relatively small reductions in ICU stay may indicate earlier stabilization, reduced exposure to ICU-specific stressors (eg, delirium), and faster transition to lower-intensity care. Previous meta-analyses showed mixed results on these outcomes,^13^, ^14^, ^15^, ^16^, ^17^^,^^19^ with reductions in LOS of up to 3 days, but with high heterogeneity in the included studies.^15^^,^^19^ Recent meta-analyses reported different results in relation to ICU stay, with some showing no effect^13^^,^^14^ and others a reduction of up to 2.22 hours.^16^ A reduction of 6 hours in ICU stay may allow for the performance of additional surgical procedures that require postoperative transfer to the ICU or the admission of emergency room patients that require access to intensive care and the necessary therapy.

Similar to our findings, prehabilitation with inspiratory muscle training reduced postprocedural pneumonia,^15^, ^16^, ^17^^,^^19^ although this was not seen with exercise-based interventions.^13^^,^^14^

Our meta-analysis found a clinically important MD of 68.87 m in 6MWD performance preoperatively in prehabilitation patients. Steinmetz et al.^13^ reported a similar improvement of 75.36 m with exercise-based prehabilitation, whereas Yau et al.^14^ found no effect due to limited data and high heterogeneity between studies.

Furthermore, meta-regression revealed significant sex differences in LOS. This is of clinical importance, as a recently published study found that females had a significantly higher risk of postoperative deep sternal wound infections (OR: 1.43; 95% CI: 1.11-1.83), prolonged LOS (MD: 1.2 days; 95% CI: 1.0−1.4), and 30-day mortality (OR: 1.76; 95% CI: 1.47−2.09) following cardiac surgery.^7^

Pooling was not possible for outcomes such as postprocedural HGS, anxiety, depression, periprocedural memory, stress, nutrition, sleep, dyspnea, angina, and long-term quality of life. GRADE assessment showed very low evidence for 6MWD at 12 weeks postprocedure and low to moderate evidence for other outcomes (Supplemental Table 4). Only 2 studies provided high-certainty evidence for the occurrence of postprocedural infections.^60^^,^^62^

RoB ranged from low to high, mainly due to poor randomization, lack of blinding in prehabilitation studies, and some concerns about the selection of the reported results. Four studies were rated with a high RoB due to unblinded assessors^32^^,^^34^^,^^36^^,^^60^ and/or because the primary outcome stated in the registry was inconsistent with the 1 mentioned in the article.^34^^,^^60^

Although catheter-based procedures were part of the search strategy, no studies examined the effect of prehabilitation before catheter-based procedures. As a result, the impact of prehabilitation before catheter-based procedures like transcatheter aortic valve replacement remains unclear. Given the high prevalence of frailty in these patients, short-term prehabilitation may be beneficial.^71^ The ongoing PERFORM-TAVR (Protein and Exercise to Reverse Frailty in OldeR Men and women undergoing Transcatheter Aortic Valve Replacement) trial is evaluating multimodal prehabilitation in this population.^72^

The expert consensus document published by Arora et al.^73^ recommended a multicomponent prehabilitation approach, including exercise, nutrition, and psychological support when needed. Of the 12 included studies incorporating more than 1 component of prehabilitation, none included all core components; most combined exercise with breathing therapy. A recently published systematic review with meta-analysis estimated the efficacy of prehabilitation components showing that patients before surgery benefit the most of exercise and nutrition as well as multicomponent interventions including exercise.^74^ Two ongoing multicenter RCTs—PRECOVERY^75^ and PRÄP-GO^76^—involving 1,822 older participants, may soon offer clinical guidance regarding multimodal prehabilitation before surgical and catheter-based procedures.

Future multicenter studies should build on previous findings, include previously unanalyzed parameters, and explore gender differences as well as differences between surgical and catheter-based procedures.

### Strengths and limitations

A rigorous search was conducted across 6 databases using broad search terms to capture all studies on prehabilitation in cardiac patients published before August 2024, following Preferred Reporting Items for Systematic reviews and Meta-Analyses guidelines. Registration in the PROSPERO database underscores the transparency of this systematic review and meta-analysis. With 21,130 titles and abstracts screened, the search was both extensive and systematic. Although catheter-based cardiac procedures were included as search terms, the final focus on prehabilitation in cardiac surgery patients ensured a targeted analysis. However, this exclusive focus may limit the generalizability of findings to broader cardiac populations.

Although safety was not part of our predefined endpoints, the available evidence indicates that structured, supervised prehabilitation carries a very low risk profile. Future trials should continue to systematically assess and report safety outcomes especially in frail patients, as these data are crucial for broader clinical implementation.

In addition, the RoB varied widely from low-to-high largely because participants and assessors could not be blinded, which may affect the objectivity of the outcomes. Robust statistical methods further strengthen the review. Nevertheless, limitations persist. High heterogeneity stemming from variations in prehabilitation interventions, durations, intensities, and modules resulted in evidence ratings of low or very low certainty. The absence of a universally accepted definition of prehabilitation results in considerable heterogeneity across studies and complicates their comparison in meta-analyses.

## Conclusions

This systematic review and meta-analysis of RCTs suggests that cardiac prehabilitation is associated with several beneficial outcomes, including improved preprocedural 6MWD, shorter ICU stay, and reduced in-hospital LOS—particularly among women. Prehabilitation was also associated with lower rates of postprocedural pneumonia. No conclusion could be drawn about the effects of the individual prehabilitation components. Importantly, the overall quality of evidence ranged from very low to moderate, and high heterogeneity across studies limits the strength of these conclusions. Future well-designed multicenter studies, employing standardized assessment tools and intervention modules, are needed to clarify these findings and establish more definitive recommendations.

Perspectives **COMPETENCY IN MEDICAL KNOWLEDGE**: Due to demographic changes, the incidence of cardiac disease and the number of patients requiring cardiac procedures are steadily increasing. Older patients face a higher risk of postprocedural complications, which can directly impact the length of hospital stay, quality of life, and physical functioning.

**COMPETENCY IN PATIENT CARE:** Prehabilitation is conducted weeks ahead of an elective cardiac procedure and involves optimizing patients` physical and mental status as well as maximizing functional reserve to improve postprocedural outcomes.

**TRANSLATIONAL OUTLOOK 1:** Prehabilitation before cardiac procedures has the potential to enhance preprocedural functional capacity and facilitate postprocedural recovery, with particularly pronounced benefits observed in women. To validate these findings and establish evidence-based recommendations, future multicenter trials employing standardized assessment tools and intervention protocols are needed.

**TRANSLATIONAL OUTLOOK 2:** The overall quality of evidence ranges from very low to moderate, and high heterogeneity across studies limits the strength of robustness of the findings. Currently, no universally accepted definition of prehabilitation has been endorsed by professional societies. This lack of consensus contributes to significant variability in study design and methodology, thereby complicating comparison and the conduct of meta-analyses.

## Funding support and author disclosures

This work was supported in part of the 10.13039/501100001659German Research Foundation, 549888584 (CS & MS), the 10.13039/501100005971German Heart Foundation (MS), the 10.13039/100000050National Heart, Lung, and Blood Institute of the 10.13039/100000002National Institutes of Health under award numbers Nation (CC), R01HL133149 (JH), the 10.13039/100000054National Cancer Institute through grant K08CA251654 (HLA), 10.13039/501100010570Ministry for Science and Culture of Lower Saxony (Niedersachsen Vorab, ZN3553) (CAFvA), and Robert-Bosch-Stiftung (32.5.1140.0007.O/MA01) (CAFvA). Prof Arnim received honoraria from serving on the scientific advisory board of Biogen, Roche, Novo Nordisk, Biontech, Lilly, RoX Health GmbH, MindAhead UG, and Dr Willmar Schwabe GmbH & Co. KG; she has received funding for travel as well as speaker honoraria from Lilly, Novo Nordisk, Roche, Novartis, Medical Tribune Verlagsgesellschaft mbH, Landesvereinigung für Gesundheit und Akademie für Sozialmedizin Niedersachsen e. V., and Dr Willmar Schwabe GmbH & Co. KG; and she has received research support from Roche diagnostics AG and research funding from the Innovationsfond (Fund of the Federal Joint Committee, Gemeinsamer Bundesausschuss, G-BA Grants No. VF1_2016-201; 01NVF21010; 01VSF21019). Prof Herrmann-Lingen is receiving royalties from Hogrefe Publishers for the German version of the Hospital Anxiety and Depression Scale and research funding from the German Ministrey of Education and Research (BMBF), the German Research Foundation (DFG), and the European Commission and he has received a lecture honorarium from Novartis. Prof Akeju is a consultant with equity in Reversal Therapeutics. Dr Hartog reports grants from “Stichting Beatrixoord Noord-Nederland” and Edwards Lifesciences SA. Prof Lee is a senior editor at the Cochrane Database of Systematic Reviews. All other authors have reported that they have no relationships relevant to the contents of this paper to disclose.
