# Supplementary material for: Prehabilitation in Patients Undergoing Cardiac Procedures: A Systematic Review and Meta-Analysis
Source: JACC Adv. 2026 Jan 29;5(3):102587. doi: 10.1016/j.jacadv.2026.102587 (PMC12874821; doi:10.1016/j.jacadv.2026.102587)

**Supplementary Online Content**

**eTable 1.** Search strategy

**eTable 2.** Definition of the clinical outcomes

**eTable 3.** Overview of the characteristics of the included studies

**eTable 4.** GRADE Summary of Quality of Evidence

**eTable 5.** Results of the meta-regression for the variables in-hospital LOS and ICU stay

**eTable 6.** PRISMA Checklist.

**eFigure 1.** Forest plot for meta-analysis of change in 6-minute walk test 4-6 weeks post-procedure in included studies

**eFigure 2.** Forest plot for meta-analysis of change in 6-minute walk test 12 weeks post-procedure in included studies

**eFigure 3.** Forest plot for meta-analysis of change in pre-procedure hand grip strength in included studies

**eFigure 4.** Forest plot for meta-analysis of change in pre-procedure overall quality of life in included studies

**eFigure 5.** Forest plot for meta-analysis of change in post-procedure overall quality of life in included studies

**eFigure 6.** Forest plot for meta-analysis of change in post-procedure anxiety in included studies

**eFigure 7.** Forest plot for meta-analysis of change in post-procedure complication delirium in included studies

**eFigure 8.** Forest plot for meta-analysis of change in post-procedure complication infection in included studies

**eFigure 9.** Bubble plot showing the association between LOS and sex

**eFigure 10**. Forest Plots of Recovery Status and Perioperative Functional Capacity Including Subgroup Analyses Uni- vs. Multimodal Prehab Programs.

**eFigure 11**. Forest Plots of Selected Postoperative Complications Including Subgroup Analyses Uni- vs. Multimodal Prehab Programs.

**eFigure 12.** Forest Plots of In-hospital Length of Stay Including Subgroup Analyses According to Prehabduration at least 2 weeks vs. less than 2 weeks.

**eFigure 13.** Forest Plots of Recovery Status and Perioperative Functional Capacity Including Subgroup Analyses Uni- vs. Multimodal Prehab Programs without breathing only or supplement/medication only studies.

**eFigure 14.** Forest Plots of Selected Postoperative Complications Including Subgroup Analyses Uni- vs. Multimodal Prehab Programs without breathing only or supplement/medication only studies.

**eFigure 15.** Forest Plots of In-hospital Length of Stay Including Subgroup Analyses According to Prehabduration at least 2 weeks vs. less than 2 weeks without breathing only or supplement/medication only studies.

**eTable 1.** Search strategy

|  | **Medline search term** |
| --- | --- |
| 1. | "Cardiac Surgical Procedures"[Mesh] OR “Cardiac surgery”[Title/Abstract] OR “Heart surgery” [Title/Abstract] OR “Cardiothoracic surgery”[Title/Abstract] OR “Cardio-thoracic surgery”[Title/Abstract] OR “Cardiac procedure*” [Title/Abstract] OR “valve surgery”[Title/Abstract] or “Valve replacement” [Title/Abstract] or “valvular replacement”[Title/Abstract] or “valve repair” [Title/Abstract] OR “valvular repair”[Title/Abstract] OR “coronary artery bypass graft*"[Title/Abstract] or CABG[Title/Abstract] OR “coronary artery bypass”[Title/Abstract] OR"Heart Valve Prosthesis Implantation"[Mesh] OR "tricuspid valve/surgery"[MeSH Terms] OR "mitral valve/surgery"[MeSH Terms] OR "pulmonary valve/surgery"[MeSH Terms] OR "aortic valve/surgery"[MeSH Terms] OR TAVR[Title/Abstract] OR TAVI[Title/Abstract] OR mitraclip[Title/Abstract] OR “transcatheter aortic valve implantation” [Title/Abstract] OR “transcatheter aortic valve replacement” [Title/Abstract] OR “transcatheter aortic”[Title/Abstract] OR “transcatheter mitral”[Title/Abstract] OR “transcatheter tricuspid”[Title/Abstract] OR “transcatheter pulmon*”[Title/Abstract] |
| 2. | "Preoperative exercise"[MeSH] OR “pre-hab*”[Title/Abstract] OR prehab*[Title/Abstract] OR prerehab*[Title/Abstract] |
| 3. | "Preoperative Care"[Mesh] OR preventative[Title/Abstract] OR pretreatment[Title/Abstract] OR “pre-treatment”[Title/Abstract] OR preventive[Title/Abstract] OR "pre-operat*"[Title/Abstract] OR "preoperat*"[Title/Abstract] OR "pre-surg*"[Title/Abstract] OR presurg*[Title/Abstract] |
| 4. | "Exercise Therapy"[Mesh] OR "Rehabilitation"[Mesh] OR Rehab*[Title/Abstract] OR Exercise[Title/Abstract] OR Physiotherapy[Title/Abstract] OR Training[Title/Abstract] OR “Physical therapy”[Title/Abstract] OR “Exercise Therapy”[Title/Abstract] OR “Exercise training”[Title/Abstract] OR “Cardiac rehab*”[Title/Abstract] OR “Resistance training”[Title/Abstract] OR “Fitness”[Title/Abstract] OR “Aerobic”[Title/Abstract] |
| 5. | "Breathing exercises"[MeSH] OR "respiratory muscle training"[Title/Abstract] OR "inspiratory muscle training"[Title/Abstract] OR “Breathing Exercise*"[Title/Abstract] OR breath*[Title/Abstract] OR respiratory[Title/Abstract] |
| 6. | "Nutrition Therapy"[Mesh] OR Nutrition*[Title/Abstract] OR Diet*[Title/Abstract] |
| 7. | "Sleep"[Mesh] or Sleep[Title/Abstract] or “Sleep Training” [Title/Abstract] |
| 8. | "Smoking Cessation"[Mesh] or Smok*[Title/Abstract] or Tobacco[Title/Abstract] |
| 9. | "Psychotherapy"[Mesh] OR "psychotherapy"[Title/Abstract] OR “Psycho-therapy”[Title/Abstract] OR "stress reduction"[Title/Abstract] OR "cognitive behavioural therapy"[Title/Abstract] OR “cognitive behavioral therapy”[Title/Abstract] OR "CBT"[Title/Abstract] |
| 10. | "Anxiety"[Mesh] OR "Stress, Psychological"[Mesh] OR Psychological[Title/Abstract] or psycho*[Title/Abstract] or Anxi*[Title/Abstract] or Stress[Title/Abstract] or Wellbeing[Title/Abstract] or “Well-being”[Title/Abstract] |
| 11. | "Cognition"[Mesh] OR cognit*[Title/Abstract] OR mental[Title/Abstract] or intellect*[Title/Abstract] OR memory[Title/Abstract] |
| 12. | "Health Education"[Mesh] or Education[Title/Abstract] or Educ*[Title/Abstract] OR "psychoeducation"[Title/Abstract] OR expectation*[Title/Abstract] |
| 13. | #1 AND #2 |
| 14. | #1 AND #3 AND (#4 OR #5 OR #6 OR #7 OR #8 OR #9 OR #10 OR #11 OR #12) |
| 15. | #13 OR #14 |

|  | **Web of Science search term** |
| --- | --- |
| 1. | TS= (Cardiac Surgical Procedure OR Cardiac surgery OR Heart surgery OR Cardiothoracic surgery OR Cardio-thoracic surgery OR Cardiac procedure* OR valve surgery or Valve replacement or valvular replacement OR valve repair OR valvular repair OR coronary artery bypass graft* or “CABG” OR coronary artery bypass OR Heart Valve Prosthesis Implantation OR tricuspid valve surgery OR mitral valve surgery OR pulmonary valve surgery OR aortic valve surgery OR “TAVR” OR “TAVI” OR “mitraclip”OR transcatheter aortic valve implantation OR transcatheter aortic valve replacement OR transcatheter aortic OR transcatheter mitral OR transcatheter tricuspid OR transcatheter pulmon*) |
| 2. | TS= (Preoperative exercise OR “pre-hab*” OR prehab* OR prerehab*) |
| 3. | TS= ( Preoperative Care OR preventative OR pretreatment OR “pre-treatment” OR preventive OR “pre-operat*” OR preoperat* OR “pre-surg*”OR presurg*) |
| 4. | TS= ( Exercise Therapy OR rehabilitation OR rehab* OR exercise OR physiotherapy OR training OR Physical therapy OR Exercise Therapy OR Exercise training OR Cardiac rehab* OR Resistance training OR fitness OR aerobic ) |
| 5. | TS= (Breathing exercises OR respiratory muscle training OR inspiratory muscle training OR Breathing Exercise* OR breath* OR respiratory) |
| 6. | TS= ( nutrition therapy OR nutrition* OR diet* ) |
| 7. | TS= ( sleep OR sleep training ) |
| 8. | TS= ( smoking cessation OR smok* OR tobacco ) |
| 9. | TS= ( psychotherapy OR stress reduction OR cognitive behavioural therapy OR cognitive behavioral therapy OR “CBT” OR “Psycho-therapy” ) |
| 10. | TS= ( anxiety OR stress OR psychological OR psycho* OR anxi* OR stress OR wellbeing OR well-being ) |
| 11. | TS= ( cogniti* OR mental OR intellect* OR memory ) |
| 12. | TS= ( health education OR educ* OR psychoeducation OR expectation ) |
| 13. | #1 AND #2 |
| 14. | #1 AND #3 AND (#4 OR #5 OR #6 OR #7 OR #8 OR #9 OR #10 OR #11 OR #12) |
| 15. | #13 OR #14 |

|  | **PsycINFO search term** |
| --- | --- |
| 1. | ‘Heart surgery’/exp OR ‘heart valve replacement’/exp OR ‘heart valve replacement’/exp OR “Cardiac surgery”:ti,ab,kw OR “Heart surgery”:ti,ab,kw OR “Cardiothoracic surgery”:ti,ab,kw OR “Cardio-thoracic surgery”:ti,ab,kw OR “Cardiac procedure*”:ti,ab,kw OR “valve surgery”:ti,ab,kw or “Valve replacement”:ti,ab,kw or “valvular replacement”:ti,ab,kw or “valve repair”:ti,ab,kw OR “valvular repair”:ti,ab,kw OR “coronary artery bypass graft*":ti,ab,kw or CABG:ti,ab,kw OR “coronary artery bypass”:ti,ab,kw OR “TAVR”:ti,ab,kw OR “TAVI”:ti,ab,kw OR “mitraclip”:ti,ab,kw OR “transcatheter aortic valve implantation”:ti,ab,kw OR “transcatheter aortic valve replacement”:ti,ab,kw OR “transcatheter aortic”:ti,ab,kw OR “transcatheter mitral”:ti,ab,kw OR “transcatheter tricuspid”:ti,ab,kw OR “transcatheter pulmon*”:ti,ab,kw |
| 2. | ‘preoperative exercise’/exp OR “pre-hab”:ti,ab,kw OR prehab*:ti,ab,kw OR prerehab*:ti,ab,kw |
| 3. | ‘preoperative period’/exp OR preventative:ti,ab,kw OR pretreatment:ti,ab,kw OR “pre-treatment”:ti,ab,kw OR preventive:ti,ab,kw OR “pre-operat*”:ti,ab,kw OR preoperat*:ti,ab,kw OR “pre-surg*”:ti,ab,kw OR presurg*:ti,ab,kw |
| 4. | 'kinesiotherapy'/exp OR 'rehabilitation'/exp OR Rehab*:ti,ab,kw OR Exercise:ti,ab,kw OR Physiotherapy:ti,ab,kw OR Training:ti,ab,kw OR “Physical therapy”:ti,ab,kw OR “Exercise Therapy”:ti,ab,kw OR “Exercise training”:ti,ab,kw OR “Cardiac rehab”:ti,ab,kw OR “cardiac rehabilitation”:ti,ab,kw OR “Resistance training”:ti,ab,kw OR Fitness OR Aerobic:ti,ab,kw |
| 5. | breathing exercise'/exp OR "respiratory muscle training":ti,ab,kw OR "inspiratory muscle training":ti,ab,kw OR “Breathing Exercise":ti,ab,kw OR “Breathing Exercises":ti,ab,kw OR breath*:ti,ab,kw OR respiratory:ti,ab,kw |
| 6. | 'diet therapy'/exp OR Nutrition*:ti,ab,kw OR Diet*:ti,ab,kw |
| 7. | 'sleep'/exp OR Sleep:ti,ab,kw OR "Sleep Training":ti,ab,kw |
| 8. | smoking cessation'/exp OR 'smoking cessation program'/exp OR Smok*:ti,ab,kw OR Tobacco:ti,ab,kw |
| 9. | 'psychotherapy'/exp OR psychotherapy:ti,ab,kw OR "stress reduction":ti,ab,kw OR "cognitive behavioural therapy":ti,ab,kw OR “cognitive behavioral therapy”:ti,ab,kw OR “CBT”:ti,ab,kw OR “Psycho-therapy”:ti,ab,kw |
| 10. | 'anxiety'/exp OR 'physiological stress'/exp OR Psychological:ti,ab,kw OR psycho*:ti,ab,kw OR Anxi*:ti,ab,kw OR Stress:ti,ab,kw OR Wellbeing:ti,ab,kw OR Well-being:ti,ab,kw |
| 11. | 'cognition'/exp OR cognit*:ti,ab,kw OR mental:ti,ab,kw OR intellect*:ti,ab,kw OR memory:ti,ab,kw |
| 12. | 'health education'/exp OR Education:ti,ab,kw OR Educ*:ti,ab,kw OR psychoeducation:ti,ab,kw OR expectation*:ti,ab,kw |
| 13. | #1 AND #2 |
| 14. | #1 AND #3 AND (#4 OR #5 OR #6 OR #7 OR #8 OR #9 OR #10 OR #11 OR #12) |
| 15. | #14 OR #15 |

|  | **Embase search term** |
| --- | --- |
| 1. | ‘Heart surgery’/exp OR ‘heart valve replacement’/exp OR ‘heart valve replacement’/exp OR “Cardiac surgery”:ti,ab,kw OR “Heart surgery”:ti,ab,kw OR “Cardiothoracic surgery”:ti,ab,kw OR “Cardio-thoracic surgery”:ti,ab,kw OR “Cardiac procedure*”:ti,ab,kw OR “valve surgery”:ti,ab,kw or “Valve replacement”:ti,ab,kw or “valvular replacement”:ti,ab,kw or “valve repair”:ti,ab,kw OR “valvular repair”:ti,ab,kw OR “coronary artery bypass graft*":ti,ab,kw or CABG:ti,ab,kw OR “coronary artery bypass”:ti,ab,kw OR “TAVR”:ti,ab,kw OR “TAVI”:ti,ab,kw OR “mitraclip”:ti,ab,kw OR “transcatheter aortic valve implantation”:ti,ab,kw OR “transcatheter aortic valve replacement”:ti,ab,kw OR “transcatheter aortic”:ti,ab,kw OR “transcatheter mitral”:ti,ab,kw OR “transcatheter tricuspid”:ti,ab,kw OR “transcatheter pulmon*”:ti,ab,kw |
| 2. | ‘preoperative exercise’/exp OR “pre-hab”:ti,ab,kw OR prehab*:ti,ab,kw OR prerehab*:ti,ab,kw |
| 3. | ‘preoperative period’/exp OR preventative:ti,ab,kw OR pretreatment:ti,ab,kw OR “pre-treatment”:ti,ab,kw OR preventive:ti,ab,kw OR “pre-operat*”:ti,ab,kw OR preoperat*:ti,ab,kw OR “pre-surg*”:ti,ab,kw OR presurg*:ti,ab,kw |
| 4. | 'kinesiotherapy'/exp OR 'rehabilitation'/exp OR Rehab*:ti,ab,kw OR Exercise:ti,ab,kw OR Physiotherapy:ti,ab,kw OR Training:ti,ab,kw OR “Physical therapy”:ti,ab,kw OR “Exercise Therapy”:ti,ab,kw OR “Exercise training”:ti,ab,kw OR “Cardiac rehab”:ti,ab,kw OR “cardiac rehabilitation”:ti,ab,kw OR “Resistance training”:ti,ab,kw OR Fitness OR Aerobic:ti,ab,kw |
| 5. | breathing exercise'/exp OR "respiratory muscle training":ti,ab,kw OR "inspiratory muscle training":ti,ab,kw OR “Breathing Exercise":ti,ab,kw OR “Breathing Exercises":ti,ab,kw OR breath*:ti,ab,kw OR respiratory:ti,ab,kw |
| 6. | diet therapy'/exp OR Nutrition*:ti,ab,kw OR Diet*:ti,ab,kw |
| 7. | 'sleep'/exp OR Sleep:ti,ab,kw OR "Sleep Training":ti,ab,kw |
| 8. | smoking cessation'/exp OR 'smoking cessation program'/exp OR Smok*:ti,ab,kw OR Tobacco:ti,ab,kw |
| 9. | 'psychotherapy'/exp OR psychotherapy:ti,ab,kw OR "stress reduction":ti,ab,kw OR "cognitive behavioural therapy":ti,ab,kw OR “cognitive behavioral therapy”:ti,ab,kw OR “CBT”:ti,ab,kw OR “Psycho-therapy”:ti,ab,kw |
| 10. | anxiety'/exp OR 'physiological stress'/exp OR Psychological:ti,ab,kw OR psycho*:ti,ab,kw OR Anxi*:ti,ab,kw OR Stress:ti,ab,kw OR Wellbeing:ti,ab,kw OR Well-being:ti,ab,kw |
| 11. | 'cognition'/exp OR cognit*:ti,ab,kw OR mental:ti,ab,kw OR intellect*:ti,ab,kw OR memory:ti,ab,kw |
| 12. | 'health education'/exp OR Education:ti,ab,kw OR Educ*:ti,ab,kw OR psychoeducation:ti,ab,kw OR expectation*:ti,ab,kw |
| 13. | #1 AND #2 |
| 14. | #1 AND #3 AND (#4 OR #5 OR #6 OR #7 OR #8 OR #9 OR #10 OR #11 OR #12) |
| 15. | #14 OR #15 |

|  | **Cochrane Central Register of Controlled Trials Library** |
| --- | --- |
| 1. | [mh "Cardiac Surgical Procedures"] OR [mh “Heart Valve Prosthesis Implantation”] OR [mh “tricuspid valve”/SU] OR [mh "mitral valve"/SU] OR [mh “pulmonary valve"/SU] OR [mh “aortic valve"/SU] OR (“Cardiac surgery” OR “Heart surgery” OR “Cardiothoracic surgery” OR “Cardio-thoracic surgery” OR “Cardiac procedure” OR “valve surgery” OR “Valve replacement” OR “valvular replacement” OR “valve repair” OR “valvular repair” OR “coronary artery bypass graft" OR “coronary artery bypass grafting" OR “CABG” OR “coronary artery bypass” OR “TAVR” OR “TAVI” OR “Mitraclip” OR "transcatheter aortic" OR "transcatheter mitral" OR "transcatheter tricuspid" OR “transcatheter pulmonic” OR “transcatheter pulmonary”):ti,ab,kw |
| 2. | [mh "Preoperative exercise"] OR (pre-hab OR prehab* OR prerehab*):ti,ab,kw |
| 3. | [mh "Preoperative Care"] OR (preventative OR pretreatment OR pre-treatment OR preventive OR pre-operat* OR preoperat* OR pre-surg* OR presurg*):ti,ab,kw |
| 4. | [mh "Exercise Therapy"] OR [mh "Rehabilitation"] OR (Rehab* OR Exercise OR Physiotherapy OR Training OR “Physical therapy” OR “Exercise Therapy” OR “Exercise training” OR “Cardiac rehab” OR “cardiac rehabilitation” OR “Resistance training” OR Fitness OR Aerobic):ti,ab,kw |
| 5. | [mh "Breathing exercises"] OR ("respiratory muscle training" OR "inspiratory muscle training" OR “Breathing Exercise" OR “Breathing Exercises" OR breath* OR respiratory):ti,ab,kw |
| 6. | [mh "Nutrition Therapy"] OR (Nutrition* OR Diet*):ti,ab,kw |
| 7. | [mh sleep] OR (Sleep OR "Sleep Training"):ti,ab,kw |
| 8. | [mh "Smoking Cessation"] OR (Smok* OR Tobacco):ti,ab,kw |
| 9. | [mh Psychotherapy] OR (psychotherapy OR "stress reduction" OR "cognitive behavioural therapy" OR “cognitive behavioral therapy” OR “CBT” OR Psycho-therapy):ti,ab,kw |
| 10. | [mh anxiety] OR [mh "Stress, Psychological"] OR (Psychological OR psycho* OR Anxi* OR Stress OR Wellbeing OR Well-being):ti,ab,kw |
| 11. | [mh Cognition] OR (cognit* OR mental OR intellect* OR memory):ti,ab,kw |
| 12. | [mh "Health Education"] OR (Education OR Educ* OR psychoeducation OR expectation*):ti,ab,kw |
| 13. | #1 AND #2 |
| 14. | #1 AND #3 AND (#4 OR #5 OR #6 OR #7 OR #8 OR #9 OR #10 OR #11 OR #12) |
| 15 | #13 OR #14 |

|  | **Scopus search term** |
| --- | --- |
| 1. | TITLE-ABS-KEY( “Cardiac Surgical Procedure” OR “Cardiac surgery” OR “Heart surgery” OR “Cardiothoracic surgery” OR “Cardio-thoracic surgery” OR “Cardiac procedure*” OR “valve surgery” or “Valve replacement” or “valvular replacement” OR “valve repair” OR “valvular repair” OR “coronary artery bypass graft*” or {CABG} OR “coronary artery bypass” OR “Heart Valve Prosthesis Implantation” OR “tricuspid valve surgery” OR “mitral valve surgery” OR “pulmonary valve surgery” OR “aortic valve surgery” OR {TAVR} OR {TAVI} OR {mitraclip} OR “transcatheter aortic valve implantation” OR “transcatheter aortic valve replacement” OR “transcatheter aortic” OR “transcatheter mitral” OR “transcatheter tricuspid” OR “transcatheter pulmon*”) |
| 2. | TITLE-ABS-KEY (“Preoperative exercise” OR “pre-hab*” OR prehab* OR prerehab*) |
| 3. | TITLE-ABS-KEY ( “Preoperative Care” OR preventative OR pretreatment OR “pre-treatment” OR preventive OR “pre-operat*” OR preoperat* OR “pre-surg*” OR presurg*) |
| 4. | TITLE-ABS-KEY ( "Exercise Therapy" OR rehabilitation OR rehab* OR exercise OR physiotherapy OR training OR "Physical therapy" OR "Exercise Therapy" OR "Exercise training" OR "Cardiac rehab*" OR "Resistance training" OR fitness OR aerobic ) |
| 5. | TITLE-ABS-KEY (“Breathing exercises” OR “respiratory muscle training” OR “inspiratory muscle training” OR “Breathing Exercise*” OR breath* OR respiratory) |
| 6. | TITLE-ABS-KEY ( "nutrition therapy" OR nutrition* OR diet* ) |
| 7. | TITLE-ABS-KEY ( sleep OR "sleep training" ) |
| 8. | TITLE-ABS-KEY ( "smoking cessation" OR smok* OR tobacco ) |
| 9. | TITLE-ABS-KEY ( psychotherapy OR "stress reduction" OR "cognitive behavioural therapy" OR "cognitive behavioral therapy" OR {CBT} OR "Psycho-therapy" ) |
| 10. | TITLE-ABS-KEY ( anxiety OR stress OR psychological OR psycho* OR anxi* OR stress OR wellbeing OR "well-being" ) |
| 11. | TITLE-ABS-KEY ( cogniti* OR mental OR intellect* OR memory ) |
| 12. | TITLE-ABS-KEY ( "health education" OR educ* OR psychoeducation OR expectation ) |
| 13. | #1 AND #2 |
| 14. | #1 AND #3 AND (#4 OR #5 OR #6 OR #7 OR #8 OR #9 OR #10 OR #11 OR #12) |
| 15. | #13 OR #14 |

**eTable 2.** Definition of the clinical outcomes

| **Clinical outcome** | **Defined or assessed by** |
| --- | --- |
| Recovery status | Length of intensive care unit (ICU stay, in hours) and length of in-hospital stay (in days) |
| Complications | Cardiac complications: atrial ﬁbrillation, arrhythmias, cardiac tamponade, pericardial effusion, heart failure, myocardial infarction, stroke, hypotension  Non-cardiac complications: pneumonia, atelectasis, pleura effusion, acute kidney injury, delirium, any infection, such as chest infection, surgical site infection and septicaemia  Mortality: in-hospital and all-cause mortality (30 and 90 days plus follow-up) |
| Functional capacity | 6-minute walk test |
| Quality of life | EuroQol Questionnaire (EQ-5D-5L), Short Form 12-item Health Survey Question-naire (SF-12), Short Form 36-item Health Survey Questionnaire (SF-36), MacNew questionnaire |
| Psychological outcomes | Anxiety: State Trait Anxiety Inventory (STAI A-Trait; STAI A-State), Hospital Anxiety and Depression Scale (HADS), Hamilton Anxiety and Depression Scale  Depression: HADS, Patient Health Questionnaire-4 (PHQ-4), Patient Health Questionnaire-9 (PHQ-9), Profile of Mood States (POMS), Beck's Depression Inventory (BDI-II), Cardiac Depression Scale (CDS), Geriatric Depression Scale (GDS)  Stress: blood cortisol level  Expectation: Treatment Expectation Questionnaire (TEX-Q) |
| Sleep quality | Sleep apnea–hypopnea syndrome, Pittsburgh Sleep Quality Index (PSQI) |
| Frailty | Clinical Frailty Scale (CFS), Essential Frailty Toolset (EFT) |
| Muscle strength | Hand grip strength |
| Cognitive outcomes | Objective memory impairment: Montreal Cognitive Assessment Test (MoCa), Mini-Mental-Status-Test (MMST)  Subjective memory impairment: Self-reported subjective memory impairment (SMI) |
| Nutrition | Mini Nutritional Assessment (MNA) |
| Cardiac-related symptoms | Dyspnea or angina pectoris measured by New York Heart Association (NYHA) score or Canadian Cardiovascular Society (CCS) score |

**eTable 3.** Overview of the characteristics of the included studies

| **No., Article, Year, Country,**  **Design** | **No. of**  **Arms** | **Surgery**  **Type**  (planned) | **Sample**  **Size** | **Participants**  (mean age, % female) | **Intervention** | **Kind of module, Setting, duration** | **Control** | **Outcomes,**  **No. of time points** | **Main findings** |
| --- | --- | --- | --- | --- | --- | --- | --- | --- | --- |
| 1.  Akbarzadeh et al., 2016,  Iran,  RPCT | 4 (SP, PS, SS, PP) | Elective CABG surgery | N=89  SP: n=22  PS: n=22  SS: n=22  PP: n=23 | SP: 56.9±7.5, 45%  PS: 58.7±8.5, 29%  SS: 58.6±6.4, 27%  PP: 58.6±8.3, 26% | Supplements consisting of  glutamine, L-carnitine, vitamin C/E, selenium | Nutrition,  Home-based,  7 d pre-OP and 30 d post-OP | Placebo sachets  of starch powder | Glucose, insulin level,  HbA1c, insulin resistance/ sensitivity, surgical complications  30 days post-OP,  5 time points | - Blood glucose levels were increased post-OP in the four groups (p<.001) - Sig. higher increase occurred in PP group compared to SP (p=.027), PS (p=.026), and SS (p=.004) - Sig. different wound infection rate between the four groups (p=.021): 26.1% in PP, 9.1% in SP, 4.5% in PS, 0% in SS |
| 2.  Akowuah et al.,  2023,  UK,  RCT | 2 (IG, CG) | Elective cardiac surgery  (CABG, valve or aortic  procedures, others) | N=180  IG: n=91  CG: n=89 | 64.7±10.2, 18% | Supervised exercise program,  2x60 min. sessions per week plus unsupervised home-based  exercise program 45 min. daily and high intensity inspiratory muscle training with inspirometer device twice-daily until the day of surgery | Exercise and breathing therapy,  Combination of supervised  inpatient and  unsupervised  home-based,  4 weeks | SMC | 6MWD, MIP,  HGS, EQ-5D-5L, HADS, ICU stay, in-hospital LOS incidence of post-OP surgical/ pulmonary  complications up to 12 weeks post-OP, AE during intervention period,  4 time points | - The 6MWT between baseline and pre-op assessment did not differ between groups - A subgroup analysis showed sig. improvements pre-OP in sarcopenic participants in 6MWD (p=.004) - Change in MIP from baseline   to all time-points was sig. greater in IG   - Of 71 pre-OP AE, six were related to prehab |
| 3.  Argunova et al.,  2022,  Russia,  RCT | 2 (IG, CG) | Elective CABG surgery | N=78  IG: n=43  CG: n=35 | IG: 61.5, 0%  CG: 63.0, 0% | Daily tread-mill training at 80%  VO_2peak_ over 40 min., incl. 5 min. warm-up/cool-down, Borg Scale: 12-15 | Exercise, Drug therapy according to clinical recommendations, correction of modifiable RF, treatment for concomitant pathologies, inpatient, 5-10 days | SMC | Frequency and type of complications, serum levels of ADMA and ET-1, combined endpoint  of total number of complications and  incidence of most life-threatening  conditions,  3 time points | - Sig. lower incidence of post-OP complications during in-hospital LOS (p=.013) in IG - 1.8 times higher ADMA levels in CG after training program compared to IG (p=.001) - Prehab reduced the likelihood of complications nine times   (OR: 0.11; 95% CI: 0.02–0.83; p=.03)   - Type 2 diabetes increased the probability of complications   by 12 times (OR: 12.3; 95% CI: 1.24–121.5; p=.03), as well as elevating the concentration of ET-1 on the eve of surgery (OR: 10.7; 95% CI: 1.4–81.3; p=.02) |
| 4.  Arthur et al.,  2000,  Canada,  RCT | 2 (IG, CG) | Elective CABG surgery | N= 46  IG: n=123  CG: n=123 | IG: 61.8, 12.2%  CG: 63.8, 17.1% | Exercise training twice per week over 90 min., aerobic interval training/stretching, intensity  at 40-70% of functional capacity, education and reinforcement at baseline and 1 week be-fore surgery, monthly nurse-initiated calls | Exercise, Education,  Psychological intervention, supervised in hospital  environment,  8 weeks | SMC | ICU stay, in-hospital LOS, peak exercise  performance, SF-36, Interpersonal Support Evaluation List, Support Evaluation List,  STAI-trait,  utilization of health care services, mortality,  4 time points | - Patients in IG spent sig. 1 day less in hospital (p=.002)   and sig. less time in the ICU (-2.1 hours; p=.001)   - Patients in IG had a better QoL during waiting period than CG (p=.004); Improved QoL continued up to 6 months after surgery (p<.001) - Mortality rates did not differ |
| **No., Article, Year, Country,**  **Design** | **No. of**  **Arms** | **Surgery**  **Type**  (planned) | **Sample**  **Size** | **Participants**  (mean age, % female) | **Intervention** | **Kind of module, Setting, duration** | **Control** | **Outcomes,**  **No. of time points** | **Main findings** |
| 5.  Auer et al.,  2017,  Germany,  RCT | 3 (IG Expect, IG Support, CG) | Elective CABG or CABG  combined with valve surgery (on-pump) | N=124  IG Expect: n=39  IG Support: n=41  CG: n=44 | IG Expect:  65.8, 12.2%  IG Support:  64.6, 18.9%  CG:  67.1, 13.5% | Two psycho-logical interventions each sessions of 50 min. and two 20 min. calls between baseline and surgery, brief psychological pre-OP intervention to optimize outcome expectations (IG Expect), or a psychological control intervention focusing on emotional support and general ad-vice but not on expectations (IG Support) | Psychological Intervention, clinical setting/home-based, supervised, duration not specified | SMC | ICU stay, in-hospital LOS, Pain Disability  Inventory six months post-OP,  2 time points | - Patients in both IGs spent sig. less days in hospital in comparison to CG (p=.028) - No sig. effect of the intervention on time spent in the ICU (p=.066) - Sig. linear trend showing that the more specific the intervention, the shorter the patient´s in-hospital LOS (p=.009) |
| 6.  Dao et al.,  2011,  USA,  RCT | 2 (TAU,  CBT) | Elective CABG  surgery | N=100  TAU: n=50  CBT: n=50 | TAU:  64.2±11.9, 35.8%  CBT:  62.8±11.8, 22.9% | MADES Therapy, 4 sessions each of 60 min.; First two sessions focused on psychoeducation about CAD, surgery, depression/anxiety, CBT, behavioral goals and cognitive strategies occurred before surgery; Second two sessions focused on reviewing cognitive strategies and distortions, as well as generating a plan for a continued change | Psychological intervention, setting not specified, duration at least 7 days before surgery | SMC | In-hospital LOS, BDI-II score, STAI-trait scores, SF-12,  3 time points | - TAU patients stayed sig. longer in the hospital than CBT patients (7.9 days vs. 9.2 days; p=.049) - Depressive symptoms increased at time of hospital discharge for the TAU group, whereas the CBT group had a decrease in depressive symptoms at the time of discharge - QoL and anxiety symptoms improved in both groups at 3 to 4 weeks with greater improvements in CBT group |
| 7.  Devecel et al.,  2018,  Turkey,  RCT | 2 (IG, CG) | Elective open-heart surgery | N=48  IG: n=24  CG: n=24 | IG:  58.0±8.7, 45.0%  CG:  59.7±7.7, 29.0% | Breathing exercises with spirometry,15 min. theoretical and practical Training when patients were admitted to the clinic, all patients received a Patient Education Booklet, patients were visited every two days and their practices were checked | Breathing therapy, un-supervised, clinical setting,  duration at least 7 days before surgery | SMC | Post-OP atelectasis, duration of extubation, ICU stay, in-hospital LOS, peripheral oxygen saturation,  4 time points | - The proportion of atelectasis development on the post-op 0th day was 71% in the IG and 92% in the CG - Patients in IG had sig. higher oxygen saturation than patients in the CG (p<.05) - Sig. lower duration of ICU stay in IG than CG (p=.024) |
| 8.  Elmarakby,  2016,  USA  RCT  (pilot study) | 2 (IG, CG) | Elective CABG surgery | N=33  IG: n=17  CG: n=16 | IG: 56.9±3.8, 0%  CG: 57.0±3.8, 0% | Customary physical therapy care one day prior to surgery and post-OP plus threshold IMT pre- and post-OP over 15 min. twice daily and at ICU 30 deep breaths twice daily with a resistance equal to 30% of MIP, individual modification of work load based on Borg CR10 scale | Breathing therapy, supervised, clinical setting, duration at least 10 to 14 days before surgery | Received customary physical therapy care one day prior to surgery and post-OP | MIP, oxygen saturation, post-OP atelectasis,  4 time points | - Before hospital discharge, the MIP and SpO2 in IG were sig. higher in IG than in CG (p<.001) - In IG the A-a gradient was sig. lower than that in CG immediately after surgery and after 40 hours (p<.001) |
| 9.  Furon et al.,  2024,  France,  RCT | 2 (IG, CG) | Elective valve replacement surgery | N=30  IG: n=15  CG: n=15 | IG:  69.7±4.8, 15.0%  CG:  68.7±7.5, 16.7% | High intensity IMT, patients completed 5 sets of 10 repetitions with one min. rest between each set twice a day at 80% of MIP, patients received face-to-face instruction and then practiced at home every day until 2 days before surgery, adherence was assessed by weekly calls and training logs | Breathing therapy, un-supervised, home-based,  3-6 weeks | Low intensity IMT at  15% of maximal MIP | 24 hour pre-OP plasma sTNFR-1 level, peri-OP inflammatory response (TNFalpha, IL-6, 8, 10, 1Beta), pulmonary function tests (VC and FEV1), and MIP duration of surgery, ICU stay, in-hospital LOS, mechanical ventilation support, time on O_2_, CRP levels, and peri-OP complications,  5 time points | - The median training duration was 34 (28-44) days - After training, the median predicted MIP was sig. higher   in the IG compared to CG (p=.04)   - sTNFR1 cytokine level in-creased sig. during training in IG, pre versus post training (p=.03) - The 24-h post-OP global inflammatory score was sig. lower in the IG than in the CG (p=.04) |

| **No., Article, Year, Country,**  **Design** | **No. of**  **Arms** | **Surgery**  **Type**  (planned) | **Sample**  **Size** | **Participants**  (mean age, % female) | **Intervention** | **Kind of module, Setting, duration** | **Control** | **Outcomes,**  **No. of time points** | **Main findings** |
| --- | --- | --- | --- | --- | --- | --- | --- | --- | --- |
| 10.  Gilani et al.,  2016,  Pakistan,  RCT | 2 (IG, CG) | Elective CABG surgery | N=170  IG: n=85  CG: n=85 | IG:  51.3±5.7, 34.1%  CG:  50.5± 6.0, 32.9% | Breathing exercise using a spirometry, incentive spirometry was done 3 times for a period of 10 min. every day before surgery | Breathing therapy, un-supervised, clinical setting,  1 week | SMC | Post-OP atelectasis, CPB time, X-clamp (cross clamp) time, ventilation time;  1 time point | - Ventilation time was sig. less in IG compared to CG (p=.05) - Sig. less post-OP atelectasis in IG compared to CG (14.1% versus 27.1%, p=.04) |
| 11.  Goodman et al.,  2008,  UK,  RCT | 2 (IG, CG) | Elective CABG surgery with or  without valve surgery | N=188  IG: n=94  CG: n=94 | IG: 63.7, 23.4%  CG: 65.9, 14.0% | Nurse-led program so-called ‘Fit For Surgery’ program, provided lifestyle counselling and prepa ration for surgery at monthly intervals, patients got a copy of the ‘Fit For Surgery’ manual | Education, reduction  of risk factors, un-supervised, home-based, monthly calls by cardiac homecare nurse, up to 9 months | SMC | Anxiety (HADS), blood pressure, cholesterol, in-hospital LOS, BMI, costs, change in smo-king rate, blood glucose, QoL (CROQ and SF-36), post-OP complications such as arrhythmia or infection,  5 time points | - For both groups blood pressure and total cholesterol sig.   improved (all, p<.03) without sig. differences between the groups   - Sig. less total costs in IG due to fewer admissions (p=.002) |
| 12.  Haghjooy Javanmard et al., 2013,  Iran,  RPCT | 2 (MG,  PL) | Elective CABG surgery | N=30  MG: n=15  PL: n=15 | MG:  58.1±9.8, 6.7%  PL:  60.1±9.2, 20% | Received 10 mg oral Melatonin before sleeping | Supplement, unsupervised, home-based, 1 month prior to surgery | Received placebo before sleeping | Nrf2 activity, Cross-clamp time, CPB time, PO_2_, O_2_ saturation, PCO_2_ ,  5 time points | - Melatonin administration was associated with a sig. increase in both plasma levels of Melatonin and Nrf2 concentration in MG compared to PG (p<.05) - Cross-clamp time, CPB time, PO_2_, O_2_ saturation, PCO_2_ all did not show any sig. differences between both groups |
| 13.  Hart,  1980,  USA,  RCT | 2 (IG, CG) | Elective CABG surgery | N=40  IG: n=20  CG: n=20 | not specified | Prerecorded 20-min. hypnotic treatment procedure incl. suggestions of relaxation, quick recovery, and visual imagery of successful post-OP recovery, each patient received a total of 5 spaced pre-OP tape-recorded hypnotic sessions; cassette tape containing  10 min. introduction to the benefits of hypnotic relaxation, upon their entrance into the hospital the IC received the same general verbal and written info pre-OP from the nursing staff as the CG | Relaxation,  home-based,  setting and duration not specified | At hospital ad-mission CG received limited, general verbal and written information pre-OP from the nursing staff (*details see Hart et al*.) | Relaxation ratings measured by daily sphygmomanometer recordings of diastolic and systolic blood pressure, recovery ratings measured by the total units of blood post-OP, STAI, patient locus of control ratings measured by IPC scales, 10 time points | - Diastolic blood pressure over the first 3 days of hospitalization in IG, was sig. lower compared to the CG values (p<.05) - Sig. reduced volume of blood required in IG post-OP compared to CG (p<.05) - Patients of IG showed sig. reduced state of anxiety and less transitory emotional states (p<.02) - The IPC Scales noted no sig. differences between both groups for the Powerful Others and Chances Scales but the Internal Scale showed sig. better values in IG than in CG (p<.01) |
| 14.  Herdy et al.,  2008,  Brazil,  RCT | 2 (IG, CG) | Elective CABG surgery | N=56  IG: n=29  CG: n=27 | IG: 61±10, 31.0%  CG: 58±9, 26.0% | Multimodal intervention consisting of progressive exercises as done in Phase I CR; respiratory exercises using spirometer and positive pressure breathing, exercises progressed from expenditure of 2 to 4 METs | Breathing therapy, Exercise (mobilization, ADL training), supervised, clinical setting, at least 5 days prior to surgery | SMC | ICU stay, days on the ward after ICU until discharge form hospital, post-OP pneumonia, atrial fibrillation or flutter detection, time until tracheal tube removal, pleural effusion, atelectasis, change in peak flow, changes in 6MWT between enrollment and discharge,  1 time point | - IG had sig. shorter duration of in-hospital LOS (p=.01), sig. lower incidence of pneumonia (p=.01) and atrial fibrillation (p=.03) compared to CG - IG had sig. lower incidence of pleural effusion (p=.03), atelectasis (p=.03) and sig. shorter time to extubation (p=.04) than the CG |
| **No., Article, Year, Country,**  **Design** | **No. of**  **Arms** | **Surgery**  **Type**  (planned) | **Sample**  **Size** | **Participants**  (mean age, % female) | **Intervention** | **Kind of module, Setting, duration** | **Control** | **Outcomes,**  **No. of time points** | **Main findings** |
| 15.  Hulzebos et al.,  2006a, Netherlands,  RCT | 2 (IG, CG) | Elective CABG surgery | N=279  IG: n=140  CG: n=139 | IG: 66.5, 22.3%  CG: 67.3, 21.9% | IG received 7 times a week individually tailored exercises incl. IMT, incentive spirometry, education on active cycle breathing, and forced expiration techniques, each session consisted of 20 min. of IMT, patients started breathing at a resistance equal to 30% of their MIP, resistance was increased incrementally, based on the rate on Borg scale, documentation of the pre-OP breathing exercise in a patient diary | Breathing therapy,  Supervised once a week,  home-based,  for at least 2  weeks prior  to surgery | SMC | Incidence of post-OP pulmonary  complications with grade 1-4 scoring: 1) cough, dyspnea, grade 2) productive cough, bronchospasm, atelectasiss, hypercarbia, grade, 3) pleural effusion, pneumonia, pneumothorax, reintubation, grade 4) ventilatory failure, ventilatory dependence >48 hours; in-hospital LOS, MIP,  2 time points | - IG had a sig. reduction in post-OP pulmonary complications (post-OP pulmonary complications grade ≥ 2, p=.02) - IG stayed in hospital one day less than the CG (p=.02) - Sub-analysis of smokers versus non-smokers showed a sig. effect of the pre-OP intervention only in smokers (p=.03) and not in the non-smokers (p=.10) |
| 16.  Hulzebos et al.,  2006b, Netherlands,  RCT  (pilot study) | 2 (IG, CG) | Elective CABG surgery | N=26  IG: n=14  CG: n=12 | IG: 70.1, 50.0%  CG: 70.5, 50.0% | Daily breathing exercises, 7x a week incl. 20 min. of IMT by the use of an inspiratory threshold-loading device, each session consisted of 20 min. of IMT, patients started breathing at a resistance equal to 30% of their MIP, resistance was increased incrementally, based on the Borg scale, documentation of the pre-OP breathing exercise in a patient diary | Breathing therapy,  Supervised once a week,  home-based,  for at least 2  weeks prior  to surgery | SMC | Feasibility as measured by occurrence of AE during training, participant satisfaction, compliance and motivation which they registered in diaries, effectiveness of IMT as measured by P_max_ at residual volume, incidence of post-OP pulmonary complications (Bronchitis, Pneumonia, Atelectasis) and in-hospital LOS,  2 time points | - The IMT was feasible and no AE occurred in the IC - Treatment satisfaction and motivation in IG, scored on 10-point scales, were 7.9 (9/0.7) and 8.2 (9/1.0) - Post-OP atelectasis occurred in sig. fewer patients in the IG than in the CG (p=.05) - No sig. differences between both group in term of in-hospital LOS hospital (p=.24) |
| 17.  Ibrar et al.,  2022,  Pakistan,  RCT | 2 (IG, CG) | Elective aortic or mitral valve repair / replacement for aortic or mitral regurgitation or stenosis or other cardiac valve procedure | N=40  IG: n=20  CG: n=20 | 34.6±10.9, 52.5% | General education and counseling plus diaphragmatic breathing, pursed lip breathing, deep breathing exercises, meditation, functional tasks, week 1: pursed lip breathing and diaphragmatic breathing 5 min. each once a day, week 2: 1 hour daily of deep breathing, meditation stress management, pursed lip breathing and diaphragmatic breathing for one hour with rest intervals according to patient condition plus functional task that include in and out of bed with 2-5 repetitions, 3-5 times per week | Education, Breathing  Therapy, Exercise (functional tasks), un-supervised,  home-based,  for at least 2  weeks prior  to surgery | SMC | Dyspnea assessed by BDI, tracheal extubation time, time to eye opening,  3 time points | - Pre-OP and post-OP findings showed that patients of IG performed better than patients in CG: sig. improvements in BDI values (p<.05) and sig. reduced eye-opening and tracheal extubation time (p<.05) |
| 18.  Jiang et al.,  2024,  China,  RCT  (multicenter study) | 2 (IG, CG) | Elective CABG surgery | N=208  IG: n=102  CG: n=106 | 66, 30.8%  IG: 65, 24.5%  CG: 66, 36.8% | Cognitive training via smartphone; online games designed to engage and challenge cognitive abilities incl. memory, imagination, reasoning, reaction time, attention, processing speed. Tasks and their difficulty were tailored to patients’ age and educational level with the goal of balancing cognitive stimulation and enjoyment, patients were instructed to spend a total of 10 hours on cognitive training, patients were asked to spend at least a full hour per day, over 2-3 sessions and a daily sessions should include at least 1 game from each of the 6 cognitive domains | Cognitive training, un-supervised,  home-based,  at least 10 days  prior to surgery | SMC | Occurrence of delirium during post-OP day 1 to 7 or until hospital discharge, assessed by CAM or CAM-ICU, post-OP cognitive dysfunction, delirium characteristics, all-cause mortality within 30 days post-OP,  10 time points | - IG was 57% less likely to develop delirium compared with those receiving SMC in CG (p=.007) - Sig. differences in IG compared to CG were observed in the incidence of severe delirium (p=.01), duration of delirium (p=.008) and number of delirium-positive days (p=.007) |
| **No., Article, Year, Country,**  **Design** | **No. of**  **Arms** | **Surgery**  **Type**  (planned) | **Sample**  **Size** | **Participants**  (mean age, % female) | **Intervention** | **Kind of module, Setting, duration** | **Control** | **Outcomes,**  **No. of time points** | **Main findings** |
| 19.  López-Hernández et al.,  2024,  Spain,  RCT  (secondary analysis) | 2 (IG, CG) | Elective valve surgery | N=68  IG: n=34  (AoS 20; MR 14)  CG: n=34  (AoS 20; MR 14) | IG_AoS:  72±12, 25.0%  IG_MR:  69±10, 36.0%  CG not specified | Personalized multimodal prehab pro-gram incl. HIIT, resistance strength training, promotion of physical activity and healthy lifestyle, incentive spirometer exercising, nutrition counselling, weekly mindfulness sessions (*details see López-Hernández et al*.) | Exercise (HIIT, resistance strength training), education, breathing therapy, relaxation, nutrition, supervised, hospital outpatient gym, duration of 4 to 6 weeks | SMC | Change in ET, 6MWT, HGS and STS test, physical activity level measured by the Yale Physical Activity Survey, post-OP complication,  2 time points | - At baseline, patients with AoS had better left systolic ventricular function and lower prevalence of atrial fibrillation compared to MR (p=.022 and p=.035 respectively) - Post-intervention patients with MR showed greater improvement in ET than AoS patients (101% vs. 66% increase from baseline) - Patients with severe MR involved in prehab showed sig. improvement in 6MWT (p=.016), YPAS (p=.008) or STS-test (p=.027) - Regarding patients allocated to CG, those with severe AoS remained functionally unchanged during surgery waiting-list time, whereas not prehabilitated patients with severe MR, showed a trend to functional decline during this time (ET 21% decrease from baseline, p=.003) |
| 20.  Norouzi et al.,  2022,  Iran,  RPCT | 2 (SL, PL) | Elective cardiac surgery | N=60  SL: n=30  PL: n=30 | 53.1±14.4 | 2 sachets of Heallagen (combination of 7 g l-arginine, 7 g l-glutamine, and 1.5 g daily HMB) | Supplements, un-supervised, home-based, 1 month priorto surgery | Placebo with identical appearance and taste (maltodextrin) | Inflammatory markers (interleukin-1, interleukin-6, tumor necrosis factor-alpha, high sensitivity C-reactive protein and erythrocyte sedimentation rate, counts of red blood cells, white blood cells, platelet, neutrophil, lymphocyte, serum levels of blood urea nitrogen and creatinine,  2 time points | - Subjects in SL group had sig. lower serum levels of interleukin-6 (p=.023), erythrocyte sedimentation rate (p<.01), high sensitivity C-reactive protein (p<.01), and lymphocyte number (p=.007) compared to the PL group, at end of the study |
| 21.  O’Gara et al.,  2020,  Israel,  RCT  (feasibility study) | 2 (IG, CG) | Elective cardiac surgery | N=40  IG: n=20  CG: n=20 | IG: 70±6, 30.0%  CG: 69±7, 25.0% | Cognitive training via a smartphone, patients were instructed to train for 2 separate 15-min. sessions per day, from the day of enrollment until 4 weeks after surgery incl. the immediate post-OP period | Cognitive training, at enrollment 1 supervised training session, home-based, at least 10 days prior to surgery | SMC | Feasibility was evaluated by enrollment patterns and adherence to protocol (*criteria see O´Gara et al.*), post-OP delirium (CAM, CAM-ICU), post-OP cognitive dysfunction assessed by MoCa, patient satisfaction, 11 time points | - Adherence, as a percentage of prescribed min. played, was 39%, 6%, and 19% for the pre-OP, immediate post-OP and post-discharge periods, respectively - The incidence of post-OP delirium and post-OP cognitive dysfunction in IG versus CG was not sig. different |
| 22.  Rice et al.,  1992,  USA,  RCT | 2 (Group A, Group B) | Elective CABG surgery | N=50  GA: n=25  GB: n=25 | GA:  60.4±8.3, 16.0%  GB:  60.0±7.4, 20.0% | Preadmission education with self-instructional  exercise booklet “Exercises for a Speedy Recovery”, it provided step-by-step instructions for performing coughing, deep breathing, leg movement, and ambulation exercises followed by feedback cues to help persons determine if they were doing them correctly | Education, Breathing  therapy, Exercise (ADL training), un-supervised, home-based, 6 to 10 days prior to surgery | SMC | Pre-OP and post-OP mood (mood adjective  checklist), pre-OP performance (exercise checklist regarding activities taught in booklet), post-OP pain (determined by daily pain medication use),  2 time points | - Preadmission self-instructed subjects reported sig. higher positive mood scores (p<.05), performed correctly sig. more exercise behaviors (p<.001) and required less teaching time following hospital   admission (p<.05)   - Post-OP, there were no group differences in mood states,   physical activity, analgesic use, or in-hospital LOS |
| **No., Article, Year, Country,**  **Design** | **No. of**  **Arms** | **Surgery**  **Type**  (planned) | **Sample**  **Size** | **Participants**  (mean age, % female) | **Intervention** | **Kind of module, Setting, duration** | **Control** | **Outcomes,**  **No. of time points** | **Main findings** |
| 23.  Rideout et al.,  2011,  UK,  RCT  (sub-analysis post 12 years) | 2 (IG; CG) | Elective CABG surgery | N=110  IG: n=50  CG: n=60 | not specified | Nurse-led CR in the pre-OP phase; monthly face-to-face contact with a nurse and additional calls as desired, offering motivational counselling to support lifestyle change, aimed at modifying known RF for individuals awaiting CABG surgery | Education (risk factor reduction), supervised, home-based, approximately 10 months | SMC | Survival at approximately 12 years post-OP, anxiety and depression (HADS), QoL (SF-36), during follow-up one assessment each year | - Relative risk of death associated with inclusion in the CR program was 0.814, risk of death increased with increasing pre-OP depression (RR 1.07) and anxiety (RR 1.09) |
| 24.  Rief et al.,  2017,  Germany,  RCT | 3 (IG Expect, IG Support, CG) | Elective CABG or CABG combined with valve surgery (on-pump) | N=124  IG Expect: n=39  IG Support: n=41  CG: n=44 | IG Expect:  65.8, 12.2%  IG Support:  64.6, 18.9%  CG:  67.1, 13.5% | Two psychological interventions each sessions of 50 min. and two 20 min. calls between baseline and surgery, brief psychological pre-OP intervention to optimize outcome expectations (IG Expect), or a psychological control intervention focusing on emotional support and general advice but not on expectations (IG Support) | Psychological Intervention, clinical set-ting/home-based, supervised, duration not specified | SMC | Disability 6 months after surgery (PDI), QoL (SF-12), fitness for work, physical activity (IPAQ), cardiac anxiety (CAQ), anxiety and depression (HADS), expectation (IPQ-E), interleukin-6/8, Tumor necrosis factor alpha, C-reactive protein,  2 time points | - The decrease in disability between baseline and follow-up was sig. in the IG EXPECT (p<.001) and in the IG SUPPORT (p=.01), but not in the CG group (p=.404) - Both psychological pre-OP interventions induced less pronounced increases in pro-inflammatory cytokine concentrations reflected by decreased interleukin-8 levels post-OP compared to changes in CG and lower interleukin-6 levels in patients of the IG EXPECT at follow-up - Both pre-surgery interventions were characterized by great patient acceptability and no adverse effects were attributed to them |
| 25.  Rosenfeldt et al.,  2005,  Australia,  RPCT | 2 (SL, PL) | Elective CABG surgery or valve replacement | N=121  SL: n=62  PL: n=59 | SL: 67, 24.0%  PL: 68, 15.0% | Oral co-enzyme Q10 (300 mg/d) | Supplement, un-supervised, home-based, 2 weeks prior to surgery | Placebo | Post-OP cardiac function, Troponin I release, 4 time points | - Patients receiving coenzyme Q10 had increased coenzyme Q10 levels in serum (p= .001), atrial trabeculae (p=.0001) and isolated mitochondria (p=.0002) compared with levels seen in patients receiving placebo - Mitochondrial respiration (adenosine diphosphate/ oxygen ratio) was more efficient (p=.012) and mitochondrial malondialdehyde content was lower (p=.002) with coenzyme Q10 than with placebo - After 30 min. of hypoxia in vitro, pectinate trabeculae isolated from patients receiving coenzyme Q10 exhibited a greater recovery of developed force compared with those in patients receiving placebo (p=.001) |
| 26.  Rosenfeldt et al.,  2011,  Australia,  RCT  (feasibility study) | 2 (IG, CG) | Elective CABG surgery and/or valve surgery | N=117  IG: n=60  CG: n=57 | IG: 62.5, 22.0%  CG: 68.0, 30.0% | Light physical exercise on cycle ergometry, treadmill walking and arm ergometry 2x week for 60 min., mental stress reduction therapy as outpatient sessions 4x 60 min. incl. family members, education about the effects and management of stress, active relaxation techniques such as deep breathing exercises and meditation, patients were encouraged to complete at least 30-60 min. of continuous aerobic exercise on at least 2 times during the week, after the 2 week program was completed, patients were encouraged to continue regular physical exercise at home for at least 30 min. on 4 days per week until surgery | Exercise (aerobic), relaxation, education, supervised during out-patient setting, unsupervised during home-based setting, ≥ 2 weeks prior to surgery | SMC | QoL (SF-36), in-hospital LOS, rate of post-OP atrial fibrillation,  3 time points | - Physical QoL was sig. improved in both groups 6 weeks post-OP compared to pre-treatment baseline (p<.001) but there was no sig. difference between groups overall (p=.35) and no sig. difference between the groups over time (p=.99) - IG compared to CG did not result in sig. changes in QoL, in-hospital LOS or incidence of atrial fibrillation |
| **No., Article, Year, Country,**  **Design** | **No. of**  **Arms** | **Surgery**  **Type**  (planned) | **Sample**  **Size** | **Participants**  (mean age, % female) | **Intervention** | **Kind of module, Setting, duration** | **Control** | **Outcomes,**  **No. of time points** | **Main findings** |
| 27.  Rueda-Gomariz et al.,  2024,  Spain,  RCT  (pilot study) | 2 (IG, CG) | Elective cardiac surgery | N=92  IG: n=46  CG: n=46 | 63.4±1.0, 28.3%  IG:  64.3±1.6, 26.1%  CG:  62.54±1.5, 30.4% | Breathing therapy by the use of a spirometer,  frequency of 3-7 times per day during at least 30 days, the exercises consist of taking deep breaths with the Tri-Ball-spirometer as  visual incentive in seated position and standing with a brief apnea at the end, followed by a slow passive exhalation | Breathing therapy, un-supervised, home-based,  ≥ 1 months | SMC | peri-OP variables: type of intervene-tion, time of extracorporeal circulation, time of ischemia, type of close (e.g., sternal cerclage wires), time of intubation, re-opening due to bleeding, post-OP variables: sternal dehiscence, wound infection, pleural effusion and atelectasis, sepsis, breathing infection, progress in results with spirometer, additional oxygen thru nasal cannula during hospitalization,  3 time points | - Sig. improvement in intubation time (p<.001) and the reintubation rate (p=.049) was reported in the IG compared to CG - Sig. lower oxygen consumption (p<.001), an improvement in the fulfillment of respiratory physiotherapy following surgery and a sig. shorter overall in-hospital LOS (p<.001) was observed in IG compared to CG |
| 28.  Sahar et al.,  2020,  Pakistan,  RCT | 2 (IG, CG) | Elective CABG surgery | N=60  IG: n=30  CG: n=30 | IG:  54.0±7.3, 10.0%  CG:  54.1±4.2, 26.7% | Patients received respiratory muscle training which consisted of the incentive spirometer, diaphragmatic breathing, segmental breathing movement and huff-coughing techniques | Breathing therapy,  supervised, clinical setting,  28 sessions  for >15 min. for each patient | SMC | 6MWD, heart rate after 6MWT, oxygen saturation after 6MWT, post-OP variables: ventilation duration, oxygen therapy, in-hospital LOS,  4 time points | - The pre-OP and post-OP readings showed that the IG performed sig. better than the CG in their 6MWT (p<.05) - IG had sig. shorter duration of mechanical ventilation, dependence on oxygen therapy and post-OP in-hospital LOS as compared with the CG (p<.05) |
| 29.  Sahar et al.,  2024,  Pakistan,  RCT | 2 (IG, CG) | Elective CABG surgery | N=74  IG: n=37  CG: n=37 | IG:  54.1±6.7, 10.8%  CG:  52.7±6.1, 24.3% | Resistance training with cuff weights/ dumbbells (10-15 reps for 1-3 times per day on major upper and lower limb muscle groups on a weekly basis, 40-50% 1 RM) once per week | Exercise (resistance training), supervised, clinical setting,  8 weeks | SMC | 6MWT, heart rate, oxygen saturation, NYHA score for functional classification, clinical frailty score, essential frailty toolset,  4 time points | - Sig. differences in 6MWT, oxygen saturation, and heart rate, NYHA classification, clinical frailty score, and essential frailty toolset were observed at pre-OP and post-OP time points (all: p<.05) |
| 30.  Salzmann et al.,  2017,  Germany,  RCT | 3 (IG Expect, IG Support, CG) | Elective CABG or CABG  combined with valve surgery (on-pump) | N=124  IG Expect: n=39  IG Support: n=41  CG: n=44 | IG Expect:  65.8, 12.2%  IG Support:  64.6, 18.9%  CG:  67.1, 13.5% | Two psycho-logical interventions each sessions of 50 min. and two 20 min. calls between baseline and surgery, brief psychological pre-OP intervention to optimize outcome expectations (IG Expect), or a psycho-logical control intervention focusing on emotional support and general advice but not on expectations (IG Support) | Psychological Intervention, clinical set-ting/home-based, supervised, duration not specified | SMC | Disability 6 months post-OP (PDI), post-OP plasma adrenaline, noradrenaline, cortisol levels,  2 time points | - Participation in IG EXPECT (p=.015) and IG SUPPORT therapy (p= .026) led to sig. lower post-OP adrenaline levels compared to CG - No sig. treatment effects of the pre-OP intervention for noradrenaline (p= .90) or cortisol (p=.30) |
| 31.  Sanjanwala et al.,  2019,  Canada,  RCT | 2 (IG, CG) | Elective cardiac surgery | N=33  IG: n=14  CG: n=19 | 67, 30.0%  IG: 69, 36.0%  CG: 66, 26.0% | Patients were referred to a community-based diabetes education and care program, DOP-team consists of a certified Diabetes Educator, nurse and dietitian with backup contact with an endocrinologist, the team assessed patients’ current diabetes self-management, glycemic status, nutrition status, physical activity level, other co-morbidities and barriers to healthy behaviors | Education (risk factor  reduction), supervised, clinical setting, duration not specified | SMC | DOP feasibility, rate of change in HbA1C between  the groups, Audit of Diabetes Quality of Life  Measure, Diabetes Treatment Satisfaction Questionnaire, Patient Health Questionnaire-9, QoL (SF-12, Health-Related Quality of Life Questionnaire),  3 time points | - The DOP group protocol adherence rate was 71% - There was no difference in the rate of HbA1C change between the groups over time (p=.994) |
| **No., Article, Year, Country,**  **Design** | **No. of**  **Arms** | **Surgery**  **Type**  (planned) | **Sample**  **Size** | **Participants**  (mean age, % female) | **Intervention** | **Kind of module, Setting, duration** | **Control** | **Outcomes,**  **No. of time points** | **Main findings** |
| 32.  Sawatzky et al.,  2014,  Canada,  RCT  (pilot study) | 2 (IG, CG) | Elective CABG surgery | N=17  IG: n=8  CG: n=9 | IG: 64±7, 25.0%  CG: 63±9, 14.0% | IG completed a min. of two 60-min. exercise sessions per week until surgery or for the duration of the 16-week prehab intervention, aerobic exercise intensity was at 85% of the max. oxygen consumption, intensity and duration of aerobic exercise was increased based on close communications between the healthcare providers and participants enrolled in IG, Types of exercises were prescribed by healthcare providers based on interests and abilities, which included walking, stationary cycling, light resistance exercise with body weight and resistance bands, and stretching, IG participants also attended 12 class-based education sessions concerning medication use, exercise, stress, diet, and cardio-vascular RF management | Exercise, education, supervised, outpatient clinic, at least 4 weeks prior to surgery | SMC | 6MWT, 5-m gait speed test, physical activity objectively measured by accelerometer, QoL (SF-36), PHQ-9, CAQ, CESEI,  3 time points | - Walking distance remained unchanged in the CG; whereas, the IG sig. increased their walking distance at the pre-OP and three month post-OP assessments (p<.05) - Gait speed was unchanged in the CG, but sig. improved in the IG by 27% and 33% pre-OP and three months post-OP, respectively (p<.05) - Enrollment in CR three months post-OP was sig. higher for IG participants (100%) than CG participants (43%; p<.05) |
| 33.  Shahood et al.,  2022,  Hungary,  RCT | 2 (IG, CG) | Elective open heart surgery | N=100  IG: n=46  CG: n=54 | Ages ranged from 40 and 83 years, 49.0%  IG: 37.8%  CG: 40.0% | Breathing exercises pre-OP after weaning from the ventilator: patients practiced 10 deep breaths  with an incentive spirometer; with breath holding during inspiration for 2 to 3 seconds, exhaling slowly in 5 deep breaths by incentive spirometer and coughing while exhaling in another 5 breaths (30 min. daily) | Breathing therapy,3 times supervised the other sessions were unsupervised, outpatient clinic, 1 week prior to surgery | SMC | Forced vital capacity, forced expiratory volume in the first second, oxygen saturation,  9 time points | - Post-OP improvements in lung function (p<.001) and oxygen saturation (p=.01) in IG were sig. compared to CG - The IG had a sig. shorter in-hospital LOS (p<.01) |
| 34.  Shakouri et al.,  2015,  Iran,  RCT | 2 (IG, CG) | Elective open heart surgery | N=60  IG: n=30  CG: n=30 | 81.0±9.56, 35.0%  IG: 54.4, 36.7%  CG: 59.3, 33.3% | Pre-OP physiotherapy techniques consisted of: 1. Breathing exercises of 10 deep breathing attempts, diaphragmatic breathing and pursing of the lips; 2. Instruction of flow-IS-based incentive spirometer and coughing; 3. Instruction of neck and shoulder mobilization exercises with an emphasis on thoracic ex-tension and rotation; 4. Instruction of muscular tension exercises; 5. Instruction of exercises to strengthen muscles responsible for moving the shoulders forwards and backwards | Breathing therapy,  Exercise (mobilization; strengthening), setting not specified, 2 weeks prior to surgery | SMC | LOS stay, duration of mechanical ventilation,  spirometry parameters, arterial blood gas parameters,  4 time points | - The mean differences were sig. for predicted forced vital capacity and predicted peak flow indices of spirometry indicator, PCO_2_ index and mean oxygen saturation of arterial blood gas index in both groups - IG had sig. shorter duration of ICU stay and duration of mechanical ventilation |
| 35.  Shokri et al.,  2022,  Egypt,  RPCT | 2 (SL, PL) | Elective CABG surgery | N=80  SL: n=40  PL: n=40 | SL:  58.4±4.4, 55.0%  PL:  60.1±4.8, 37.5% | Patients received a single intravenous dose of ferric carboxymaltose (1000 mg in 100 mL saline) infused for 15 min. | Supplement, supervised, clinical setting, 7 days prior  to surgery | Patients received a single‑dose infusion of 100 mL saline (sodium chloride 9 mg/ mL) for 15 min. | Incidence of anemia, hemoglobin level, number of packed red blood cell units, percentage of reticulocytes, in-hospital LOS, ICU stay, incidence of post-OP complications including cerebrovascular stroke, prolonged ventilation, heart failure, cardiac tamponade, hospital mortality, infection (sepsis and pneumonia), myocardial infarction, peri-cardial effusion,  5 time points | - Sig. reduced incidence of anemia 4 weeks post discharge in SL compared to CG (32% versus 80%, p<.001) - Sig. decrease in in-hospital LOS (p<.001) and ICU stay (p<.001) in SL compared to CG - Sig. less transfusion of packed red blood cells post-OP in SL compared to CG (12.5% vs 55%, p<.001) - Sig. lower reticulocyte count post-OP and 1 week after discharge in SL compared to CG (p<.001) |
| **No., Article, Year, Country,**  **Design** | **No. of**  **Arms** | **Surgery**  **Type**  (planned) | **Sample**  **Size** | **Participants**  (mean age, % female) | **Intervention** | **Kind of module, Setting, duration** | **Control** | **Outcomes,**  **No. of time points** | **Main findings** |
| 36.  Sowade et al.,  1998,  Germany.  RPCT | 2 (SL, PL) | Elective open heart surgery | N=76  SL: n=38  PL: n=38 | not specified | Five gifts of 500U epoitin beta/kg, 4 in the 14 days pre-OP; all patients received 300 mg Fe2+  orally per day | Supplements, supervised, clinical setting, 14 days prior to surgery | Placebo, 4 in the 14 days before surgery; all patients received 300 mg Fe2+ orally per day | Percentage of patients receiving allogenic blood transfusion, number of red blood cells, hematologic parameters, iron metabolism, blood pressure, AEs, different lab results,  4 time points | - SL therapy produced sig. increases in hemoglobin concentration, reticulocyte count, hematocrit, the hypo-chromic red blood cells and a decrease in transferrin saturation (41%) compared to the PL group pre-OP (all, p<.0001) - Sig. increase in pre-OP hemoglobin in SL competed to PL (p<.0001) - Sig. less patients in SL compared to PL received blood transfusion (11% vs. 53%, p=.0003) |
| 37.  Steinmetz et al.,  2020,  Germany,  RCT | 2 (IG, CG) | Elective CABG surgery | N=203  IG: n=88  CG: n=115 | 67.1±8.4 years, 10.3%  IG: 66.1±9.0  CG: 67.9±7.9 | Two-week exercise program including an individualized and monitored cycle ergometer training 3x per week, exercise intensity was 70% of VO_2peak_, every training session included 2 aerobic exercise work-outs with a 15-min. phase of light gymnastics in between, aerobic exercises started with 2x10 min. cycling workouts which were increase up to two 25 min. cycling at the end; light gymnastic pro-gram incl. breathing techniques and coordination exercises on a chair | Exercise (aerobic endurance training, co-ordination), Breathing techniques, supervised, clinical setting, at least 2 weeks prior to surgery | SMC | Pre- and post-OP exercise capacity, (CPET values), functional capacity (6MWT, TUG), QoL (MacNew Questionnaire),  4 time points | - Sig. improvement in functional capacity and quality of life pre-OP in IG compared to CG; similar effects were observed post-OP at the begin of CR (all: p≤.018) - No differences were found in the CPET values between both groups |
| 38.  Trubnikova et al.,  2021,  Russia,  RCT | 2 (IG, CG) | Elective CABG surgery | N=103  IG: n=50  CG: n=53 | IG: 59.0, 0%  CG: 58.0, 0% | Aerobic physical training on a treadmill was implemented once a day; Daily training session lasted 40 min., session included a 5-min warm-up and a 5-min cool-down period and a 30-min training phase, modified Borg scale was used to assess the load level (the optimal load level was 12–15 points), load accounted for 80% of the VO_2max_ | Exercise (aerobic endurance training), supervised,  clinical setting, at least 5 to 7 days prior to surgery | SMC | Cognitive status assessment (MMSE, FAB), extended neuropsychological testing, multichannel computed electroencephalography, blood analysis (S100b, NSE, and BDNF),  4 time points | - Post-OP cognitive dysfunction occurred in 27 patients in the IG and 39 patients in the CG (p=.029) - Patients of the CG demonstrated a higher percentage of theta1 power increase in the relative change values as compared to the IG patients (p=.015) - The short pre-OP training was associated with low plasma S100b concentration, but high BDNF levels in the post-OP period - Patients who underwent a short pre-OP training had better cognitive and electrical cortical activity indicators - Markers of the neurovascular unit indicated lower peri-OP brain injury after CABG in those who underwent training |
| 39.  Tully et al.,  2008,  Canada,  RCT  (pilot study) | 2 (IG, CG) | Elective CABG surgery | N=11  IG: n=6  CG: n=5 | IG: 70.0, 0%  CG: 57.0. 0% | 40% carbs replaced by low glycemic index foods | Nutrition, unsupervised, home-based, duration not specified | 40% carbs re-placed by high glycemic index foods | Insulin sensitivity, markers of inflammation (C-peptide, Interleucin-6),  3 time points | - In IG Glycemic index decreased by 11 units - In CG Glycemic index increased by 8.6 units - In IG pre-OP Homeostasis Model Assessment insulin sensitivity sig. improved (p=.018) |

| **No., Article, Year, Country,**  **Design** | **No. of**  **Arms** | **Surgery**  **Type**  (planned) | **Sample**  **Size** | **Participants**  (mean age, % female) | **Intervention** | **Kind of module, Setting, duration** | **Control** | **Outcomes,**  **No. of time points** | **Main findings** |
| --- | --- | --- | --- | --- | --- | --- | --- | --- | --- |
| 40.  Turky et al.,  2017,  Egypt,  RCT | 2 (IG, CG) | Elective CABG surgery | N=40  IG: n=20  CG: n=20 | IG: 56.9 ± 3.8, 0%  CG: 57.0 ± 4.4, 0% | IMT with powerbreathe intensity at 30% MIP, 3x10 slow breaths, 2x daily | Breathing therapy, un-supervised, home-based, 10 to 14 days before surgery | SMC | Alveolar-arterial oxygen gradient, inspiratory muscle power, peripheral oxygen saturation,  5 time points | - In IG sig. alveolar-arterial O₂ gradient gains were observed (p<.05) - In IG the inspiratory muscle power improved at all time points - At discharge the inspiratory power was sig. increased to baseline |
| 41.  Ulas et al.,  2008,  Turkey,  RPCT | 2 (SL, PL) | Elective CABG surgery | N=50  SL: n=25  PL: n=25 | 18% women  SL:  44.2 ± 2.3, 16%  PL:  47.2 ± 2.8, 20% | Received ambroxol | Drug intervention, 1 week before and after CABG surgery | Received placebo | Groups compared on pulmonary function tests, lecithin / sphingomyelin (L/S) ratio in bronchoalveolar lavage fluid, arterial blood gases, and peri-OP morbidity,  2 time points | - Post-op lecithin / sphingomyelin was lower than pre-OP in both groups, no sig. group differences in pre-OP or post-OP levels - Pre-OP partial pressure of oxygen was similar; post-op sig. lower in CG (p<.05) - Post-OP forced vital capacity and forced expiratory volume in 1 second were lower than pre-OP, more in CG - Peri-OP morbidity was similar in both groups |
| 42.  Valkenet et al.,  2016, Netherlands,  RCT  (secondary analysis) | 2 (IG, CG) | Elective CABG surgery | N=235  IG: n=119  CG: n=116 | Ages ranged from 37 and 84 years, 20.9%  IG: 22.7%  CG: 18.9% | Patients received IMT, spirometry, education on breathing, coughing, and mobilization pre-OP, tailored to each, IMT training 20 min./day, 7x/ wk, stopped day before surgery | Breathing therapy, supervised once a week by physical therapists, home-based, for at least 2 weeks prior to surgery | SMC | Incidence of post-OP pneumonia, in-hospital LOS, Health-related QoL (SF-36 and EQ-5D-3L) | - IG had sig. less pneumonia and shorter in-hospital LOS - In all patients QoL improved post-OP - No QoL difference between groups over time were observed |
| 43.  Weiner et al.,  1998,  Israel,  RCT | 2 (IG, CG) | Elective CABG surgery | N=84  IG: n=42  CG: n=42 | 29.5% women  IG: 59.2±3.8  CG: 63.8±3.1 | IG trained daily, 6 days/ week, for 2-4 weeks, each session was 30 min. with supervision,  a inspiratory muscle trainer was used with  resistance started at 15-60% of PI_max_ | Breathing therapy, supervised, home-based, 2-4 weeks prior to surgery | CG per-formed same training concept as IG but breathed through the device without resistance | FVC, FEV1, arterial blood gases (pH, PaO_2_, PaCO_2_), PI_max_ at residual volume, peak pressure at 60s load,  3 time points | - CG showed decreased muscle, lung, and gas function - IG had stable parameters post-OP - 26% of CG, 5% of IG needed ventilation >24h |
| 44.  Westhuyzen et al.,  1997,  Australia,  RPCT | 2 (SL, PL) | Elective CABG surgery | N=76  SL: n=38  PL: n=38 | 59.9±7.3, 17.1%  SL:  60.0±7.0, 13.2%  PL:  59.7±7.7, 21.1% | Subjects were given capsules that contains of  750 IU Vitamin E, 12 hours pre-OP 1g Vita-min C was given | Supplements, unsupervised, home-based, 10 days prior to surgery | Placebo contains of sucrose | CK-MB, plasma tocopherol, QRS score (54 criteria), T1-201 SPECT scans,  2 time points | - Plasma tocopherol rose fourfold, then fell 70% drop in supplement; negligible placebo - Supplements prevented depletion of plasma antioxidant but did not reduce injury |

**Abbreviations.** 1RM, One Repetition Maximum; 6MWD, 6-minute walk distance; 6MWT, 6-minute walk test; ADL, activity of daily living; ADMA, asymmetric dimethylarginine; AE, adverse event; AoS, aortic stenosis; BDI-II score, Beck Depressions-Inventar II score; BDI, baseline dyspnea index; BDNF, brain-derived neurotrophic factor; BMI, Body Mass Index; CABG, Coronary artery bypass surgery; CBT, cognitive behavioral therapy; CAD, coronary artery disease; CAM, Confusion Assessment Method; CAM-ICU, Confusion Assessment Method for Intensive Care Units; CAQ, cardiac anxiety questionnaire; CESEI, Cardiac Exercise Self-Efficacy Instrument; CG, control group; CPB time, cardiopulmonary bypass time; CPET, Cardio Pulmonary Exercise Test; CR, cardiac rehabilitation; CROQ, Coronary Revascularization Outcome Questionnaire; CRP levels, c-reactive protein; d, days; DOP, Diabetes Optimization Program; EF, ejection fraction; ET, endurance time; ET-1, endothelin-1; EQ-5D-5L, European Quality of Life 5 Dimensions 5 Level Version questionnaire; Expect, expectation manipulation intervention; FAB, Frontal Assessment Battery; FEV1, Forced expiratory volume in the first second of expiration; HADS, Hospital Anxiety and Depression Scale; HGS, hand grip strength; HIIT, high-intensity interval training; ICU, intensive care unit; IMT, inspiratory muscle training; incl., inclusive; IPAQ, International Physical Activity Questionnaire; IPQ-E, Illness Perception Questionnaire Expectation; IG, intervention group; LOS, length of stay; IPC, interpersonal circumplex; RPCT, randomized placebo-controlled trial; MADES, Managing Anxiety and Depression using Education and Skills; METs, metabolic equivalents; MG, melatonin group; MIP, maximal inspiratory pressure; min., minute; MMSE, Mini-Mental-Status-Test; MoCa, Montreal Cognitive Assessment Test; MR, mitral regurgitation; Nrf2, Nuclear erythroid 2-related factor 2; NSE, Neuron Specific Enolase; NYHA, New York Heart Association; PL, placebo group; S100b, S100 calcium-binding protein B; Sig., significant; SMC, standard medical care; SP, Supplement/Placebo group; Support, supportive care therapeutic attention without targeting expectations; PS, Placebo/Supplement group; SF-12 or 36, Short Form 12 or 36 Health Survey; SS, Supplement/Supplement group; STAI-trait, State-Trait Anxiety Inventory; O2, oxygen; PDI, Pain Disability Index; PCO2, partial pressure of carbon dioxide; PHQ-9, Patient Health Questionnaire-9; PO2, oxygen partial pressure; PP, Placebo/Placebo group; RF, risk factors; STS, sit to stand test; TAU, treatment as usual; UK, United Kingdom; VC, vital capacity; VO2peak, peak oxygen uptake; QoL, Quality of Life

**eTable 4.** GRADE Summary of Quality of Evidence

**Author(s):** Carolin Steinmetz, Stephanie Heinemann

**Question:** Prehabilitation compared to Controls for patients prior to cardiac procedures

**Setting:** clinical setting

| **Certainty assessment** | | | | | | | **№ of patients** | | **Effect** | | **Certainty** | **Importance** |
| --- | --- | --- | --- | --- | --- | --- | --- | --- | --- | --- | --- | --- |
| **№ of studies** | **Study design** | **Risk of bias** | **Inconsistency** | **Indirectness** | **Imprecision** | **Other considerations** | **Prehabilitation** | **Controls** | **Relative (95% CI)** | **Absolute (95% CI)** |  |  |
| **Length of stay at intensive care unit** | | | | | | | | | | | | |
| 16 | randomised trials | serious^a^ | serious^b^ | not serious | not serious | none | 587 | 585 | - | MD **6.03 lower** (12.01 lower to 0.06 lower) | ⨁⨁◯◯ Low^a,b^ |  |
| **Length of stay in-hospital** | | | | | | | | | | | | |
| 18 | randomised trials | serious^a^ | serious^b^ | not serious | not serious | none | 772 | 759 | - | MD **0.95 lower** (1.77 lower to 0.13 lower) | ⨁⨁◯◯ Low^a,b^ |  |
| **6-minute walk distance (post-procedural prior to hospital discharge)** | | | | | | | | | | | | |
| 4 | randomised trials | serious^a^ | not serious^c^ | not serious | not serious | none | 184 | 209 | - | MD **49 higher** (10.68 lower to 108.68 higher) | ⨁⨁⨁◯ Moderate^a,c^ |  |
| **6-minute walk distance (4-6 weeks post-procedural)** | | | | | | | | | | | | |
| 2 | randomised trials | serious^d^ | not serious^c^ | not serious | not serious | none | 179 | 204 | - | MD **25.31 higher** (162.69 lower to 213.32 higher) | ⨁⨁⨁◯ Moderate^c,d^ |  |
|  | | | | | | | | | | | | |
|  | | | | | | | | | | | | |
| **6-minute walk distance (12 weeks post-procedural)** | | | |  |  |  |  |  |  |  |  |  |
| 2 | randomised trials | very serious^e^ | serious^b^ | not serious | not serious | none | 99 | 96 | - | MD **59.9 higher** (766.89 lower to 886.7 higher) | ⨁◯◯◯ Very low^b,e^ |  |
| **Quality of life (4-6 weeks post-procedural)** | | | | | | | | | | | | |
| 3 | randomised trials | serious^d^ | not serious^f^ | not serious | not serious | none | 227 | 253 | - | SMD **0.07 SD lower** (0.5 lower to 0.36 higher) | ⨁⨁⨁◯ Moderate^d,f^ |  |
| **Anxiety (post-procedural)** | | | | | | | | | | | | |
| 2 | randomised trials | serious^a^ | not serious^c^ | not serious | not serious | none | 68 | 69 | - | SMD **1.1 SD lower** (4.54 lower to 2.34 higher) | ⨁⨁⨁◯ Moderate^a,c^ |  |
| **Complication_atelectasis (post-procedural)** | | | | | | | | | | | | |
| 5 | randomised trials | serious^a^ | serious^b^ | not serious | not serious | none | 25/132 (18.9%) | 60/129 (46.5%) | **OR 0.22** (0.03 to 1.60) | not estimable | ⨁⨁◯◯ Low^a,b^ |  |
| **Complication_atrial_fibrillation (post-procedural)** | | | | | | | | | | | | |
| 4 | randomised trials | serious^a^ | not serious^c^ | not serious | not serious | none | 31/103 (30.1%) | 35/96 (36.5%) | **OR 0.69** (0.07 to 7.34) | not estimable | ⨁⨁⨁◯ Moderate^a,c^ |  |
| **Complication_pleural_effusion (post-procedural)** | | | | | | | | | | | | |
| 4 | randomised trials | serious^a^ | serious^b^ | not serious | not serious | none | 28/142 (19.7%) | 53/140 (37.9%) | **OR 0.49**  (0.07 to 3.52) | not estimable | ⨁⨁◯◯ Low^a,b^ |  |
| **Complication_delirium (post-procedural)** | | | | | | | | | | | | |
| 2 | randomised trials | serious^g^ | not serious^c^ | not serious | not serious | none | 33/122 (27.0%) | 49/126 (38.9%) | **OR 0.78** (0 to 2537.41) | not estimable | ⨁⨁⨁◯ Moderate^c,g^ |  |
| **Complication_infection (post-procedural)** | | | | | | | | | | | | |
| 2 | randomised trials | not serious | not serious^f^ | not serious | not serious | none | 4/65 (6.2%) | 8/65 (12.3%) | **OR 0.47** (0 to 1720.13) | not estimable | ⨁⨁⨁⨁ High^f^ |  |
| **all cause mortality** | | | | | | | | | | | | |
| 5 | randomised trials | serious^a^ | not serious | not serious | not serious | none | 33/381 (8.7%) | 45/389 (11.6%) | **OR 0.82** (0.27 to 2.46) | not estimable | ⨁⨁⨁◯ Moderate^a^ |  |
| **Complication_pneumonia (post-procedural)** | | | | | | | | | | | | |
| 5 | randomised trials | serious^d^ | not serious^f^ | not serious | not serious | none | 18/372 (4.8%) | 53/357 (14.8%) | **OR 0.33**  (0.15 to 0.72) | not estimable | ⨁⨁⨁◯ Moderate^d,f^ |  |
| **pre-procedural 6MWD** | | | | | | | | | | | | |
| 6 | randomised trials | serious^a^ | serious^h^ | not serious | not serious | none | 288 | 312 | - | MD **68.87 higher** (12.76 higher to 124.98 higher) | ⨁⨁◯◯ Low^a,h^ |  |
|  | | | | | | | | | | | | |
| **pre-procedural QoL** | | | | | | | | | | | | |
| 2 | randomised trials | serious^d^ | not serious | not serious | not serious | none | 179 | 204 | - | SMD **0.11 SD higher** (1.18 lower to 1.39 higher) | ⨁⨁⨁◯ Moderate^d^ |  |
| **pre-procedural hand grip strength** | | | | | | | | | | | | |
| 2 | randomised trials | serious^a^ | not serious | not serious | not serious | none | 125 | 123 | - | MD **2.35 higher** (0.89 lower to 5.58 higher) | ⨁⨁⨁◯ Moderate^a^ |  |

**CI:** confidence interval; **MD:** mean difference; **OR:** odds ratio; **SMD:** standardised mean difference

#### Explanations

a. Some concerns with bias in randomization process, blinding of patients/assessors and blinding of measurement of the outcome.; b. statistical analysis showing sig. heterogeneity and visual inconsistency; c. moderate heterogeneity not significant; d. Some concerns with bias in measurement of the outcome and blinding of patients; e. high bias in measurement of the outcome and blinding of patients; f. low heterogeneity not significant; g. Bias due to randomization process and deviations from intended intervention; h. high and sig. heterogeneity (I²>70%)

**eTable 5.** Results of the meta-regression for the variables in-hospital LOS and ICU stay

For categorical covariables (like type of intervention), the intercept constitutes a reference, and the remaining coefficients refer to differences to this category.

**In-hospital LOS:**

Type of intervention:

| Coefficient | Estimate, SE, 95% CI | p-value |
| --- | --- | --- |
| Intercept (Breathing therapy) | -0.83, (0.75), [-2.44; 0.78] | 0.29 |
| Multimodality | -0.13, (1.00), [-2.29; 2.02] | 0.90 |
| Cognitive training or Drug intervention or Nutrition | 1.32, (1.26), [-1.40; 4.04] | 0.31 |
| Psychological intervention | -1.54, (1.53), [-4.85; 1.77] | 0.33 |
| Supplement | -1.10, (1.32), [-3.96; 1.75] | 0.42 |

Mean age:

| Coefficient | Estimate, (SE), 95% CI | p-value |
| --- | --- | --- |
| Intercept | -2.80, (4.32), [-12.01; 6.41] | 0.53 |
| Mean age | 0.03, (0.069), [-0.12; 0.18] | 0.68 |

Proportion of male:

| Coefficient | Estimate, (SE), 95% CI | p-value |
| --- | --- | --- |
| Intercept | -7.34, (2.36), [-12.38; -2.30] | **0.007** |
| Proportion of male | 8.08, (2.95), [1.80; 14.36] | **0.015** |

Location:

| Coefficient | Estimate, (SE), 95% CI | p-value |
| --- | --- | --- |
| Intercept (Africa) | -2.18, (1.06), [-4.47; 0.12] | 0.061 |
| America | 1.06, (1.27), [-1.68; 3.79] | 0.42 |
| Asia | 2.04, (1.40), [-0.9891; 5.08] | 0.17 |
| Europe | 0.81, (1.35), [-2.10; 3.72] | 0.56 |
| Pacific | 2.51, (1.53), [-0.80; 5.81] | 0.13 |

Type of procedure:

| Coefficient | Estimate, (SE), 95% CI | p-value |
| --- | --- | --- |
| Intercept | 0.32, (0.74), [-1.25; 1.89] | 0.67 |
| CABG | -1.76, (0.85), [-3.58; 0.057] | 0.057 |

Multimodality:

| Coefficient | Estimate, (SE), 95% CI | p-value |
| --- | --- | --- |
| Intercept | -0.95, (0.50), [-2.01; 0.11] | 0.076 |
| Multimodality | -0.016, (0.84), [-1.80; 1.77] | 0.99 |

Duration of prehabilitation:

| Coefficient | Estimate, (SE), 95% CI | p-value |
| --- | --- | --- |
| Intercept | -0.95, (0.50), [-2.01; 0.11] | 0.08 |
| Slope (At-least-two-weeks prehab) | -0.02, (0.84), [-1.80; 1.77] | 0.98 |

**ICU stay:**

Type of intervention:

| Coefficient | Estimate, (SE), 95% CI | p-value |
| --- | --- | --- |
| Intercept | -11.19, (5.79), [-23.80; 1.42] | 0.077 |
| Multimodality | 7.30, (7.92), [-9.94; 24.55] | 0.37 |
| Cognitive training or Drug intervention or Nutrition or Supplement | 5.63, (8.39), [-12.64; 23.90] | 0.51 |
| Psychological intervention | 8.22, (12.64), [-19.33; 35.76] | 0.53 |

Mean age:

| Coefficient | Estimate, (SE), 95% CI | p-value |
| --- | --- | --- |
| Intercept | -9.01, (36.12), [-87.70; 69.68] | 0.81 |
| Mean age | 0.033, (0.59), [-1.26; 1.33] | 0.96 |

Proportion of male:

| Coefficient | Estimate, (SE), 95% CI | p-value |
| --- | --- | --- |
| Intercept | -34.26, (15.52), [-67.79; -0.73] | **0.046** |
| Proportion of male | 35.89, (19.55), [-6.34; 78.13] | 0.090 |

Location:

| Coefficient | Estimate, (SE), 95% CI | p-value |
| --- | --- | --- |
| Intercept | -10.19, (6.43), [-24.19; 3.81] | 0.14 |
| America | 8.75, (7.52), [-7.64; 25.14] | 0.27 |
| Asia | 4.400, (8.11), [-13.28; 22.08] | 0.60 |
| Europe | -11.57, (10.69), [-34.85; 11.71] | 0.30 |

Type of procedure:

| Coefficient | Estimate, (SE), 95% CI | p-value |
| --- | --- | --- |
| Intercept | -11.12, (9.21), [-31.19; 8.94] | 0.25 |
| CABG | 5.31, (9.88), [-16.21; 26.83] | 0.60 |

Multimodality:

| Coefficient | Estimate, (SE), 95% CI | p-value |
| --- | --- | --- |
| Intercept | -7.42, (3.62), [-15.18; 0.35] | 0.060 |
| Multimodality | 3.54, (6.14), [-9.64; 16.72] | 0.57 |

Duration of prehabilitation:

| Coefficient | Estimate, (SE), 95% CI | p-value |
| --- | --- | --- |
| Intercept | -8.55, (4.62), [-18.53; 1.43] | 0.09 |
| Slope (At-least-two-weeks prehab) | 3.99, (6.36), [-9.74; 17.73] | 0.54 |

**eTable 6.** PRISMA Checklist

| **Section and Topic** | **Item #** | **Checklist item** | **Location where item is reported** |
| --- | --- | --- | --- |
| **TITLE** | | |  |
| Title | 1 | Identify the report as a systematic review. | p.1 |
| **ABSTRACT** | | |  |
| Abstract | 2 | See the PRISMA 2020 for Abstracts checklist. | p.5 |
| **INTRODUCTION** | | |  |
| Rationale | 3 | Describe the rationale for the review in the context of existing knowledge. | p.7-8 |
| Objectives | 4 | Provide an explicit statement of the objective(s) or question(s) the review addresses. | p.7-8 |
| **METHODS** | | |  |
| Eligibility criteria | 5 | Specify the inclusion and exclusion criteria for the review and how studies were grouped for the syntheses. | p.8-9 |
| Information sources | 6 | Specify all databases, registers, websites, organisations, reference lists and other sources searched or consulted to identify studies. Specify the date when each source was last searched or consulted. | p.8 |
| Search strategy | 7 | Present the full search strategies for all databases, registers and websites, including any filters and limits used. | see eTable 1 in Supplement 1 |
| Selection process | 8 | Specify the methods used to decide whether a study met the inclusion criteria of the review, including how many reviewers screened each record and each report retrieved, whether they worked independently, and if applicable, details of automation tools used in the process. | p.9, see Table 1 |
| Data collection process | 9 | Specify the methods used to collect data from reports, including how many reviewers collected data from each report, whether they worked independently, any processes for obtaining or confirming data from study investigators, and if applicable, details of automation tools used in the process. | p.9, see Figure 1 |
| Data items | 10a | List and define all outcomes for which data were sought. Specify whether all results that were compatible with each outcome domain in each study were sought (e.g. for all measures, time points, analyses), and if not, the methods used to decide which results to collect. | p.9-10, see eTable 2 in Supplement 1 |
|  | 10b | List and define all other variables for which data were sought (e.g. participant and intervention characteristics, funding sources). Describe any assumptions made about any missing or unclear information. | p.9-10, see eTable 3 in Supplement 1 |
| Study risk of bias assessment | 11 | Specify the methods used to assess risk of bias in the included studies, including details of the tool(s) used, how many reviewers assessed each study and whether they worked independently, and if applicable, details of automation tools used in the process. | p.9 |
| Effect measures | 12 | Specify for each outcome the effect measure(s) (e.g. risk ratio, mean difference) used in the synthesis or presentation of results. | p.9-10 |
| Synthesis methods | 13a | Describe the processes used to decide which studies were eligible for each synthesis (e.g. tabulating the study intervention characteristics and comparing against the planned groups for each synthesis (item #5)). | p.8-9 |
|  | 13b | Describe any methods required to prepare the data for presentation or synthesis, such as handling of missing summary statistics, or data conversions. | p.9-10 |
|  | 13c | Describe any methods used to tabulate or visually display results of individual studies and syntheses. | p.9-10 |
|  | 13d | Describe any methods used to synthesize results and provide a rationale for the choice(s). If meta-analysis was performed, describe the model(s), method(s) to identify the presence and extent of statistical heterogeneity, and software package(s) used. | p.9-10 |
|  | 13e | Describe any methods used to explore possible causes of heterogeneity among study results (e.g. subgroup analysis, meta-regression). | p.10 |
|  | 13f | Describe any sensitivity analyses conducted to assess robustness of the synthesized results. | p.10 |
| Reporting bias assessment | 14 | Describe any methods used to assess risk of bias due to missing results in a synthesis (arising from reporting biases). | p.9-10 |
| Certainty assessment | 15 | Describe any methods used to assess certainty (or confidence) in the body of evidence for an outcome. | p.9-10 |
| **RESULTS** | | |  |
| Study selection | 16a | Describe the results of the search and selection process, from the number of records identified in the search to the number of studies included in the review, ideally using a flow diagram. | p.11,  see Figure 1 |
|  | 16b | Cite studies that might appear to meet the inclusion criteria, but which were excluded, and explain why they were excluded. | p.11 |
| Study characteristics | 17 | Cite each included study and present its characteristics. | p.11-12, see eTable 3 in Supplement 1 |
| Risk of bias in studies | 18 | Present assessments of risk of bias for each included study. | p.12-13; see Figure 2 |
| Results of individual studies | 19 | For all outcomes, present, for each study: (a) summary statistics for each group (where appropriate) and (b) an effect estimate and its precision (e.g. confidence/credible interval), ideally using structured tables or plots. | p.13-15, see eFigures 1-9 and eTable 5 in Supplement 1 |
| Results of syntheses | 20a | For each synthesis, briefly summarise the characteristics and risk of bias among contributing studies. | p. 12-13, Figure 1, see eTable 3 in Supplement 1 |
|  | 20b | Present results of all statistical syntheses conducted. If meta-analysis was done, present for each the summary estimate and its precision (e.g. confidence/credible interval) and measures of statistical heterogeneity. If comparing groups, describe the direction of the effect. | p.13-15, see Figure 3-4, eFigures 1-9 and eTable 5 in Supplement 1 |
|  | 20c | Present results of all investigations of possible causes of heterogeneity among study results. | p.13-15 |
|  | 20d | Present results of all sensitivity analyses conducted to assess the robustness of the synthesized results. | N/A |
| Reporting biases | 21 | Present assessments of risk of bias due to missing results (arising from reporting biases) for each synthesis assessed. | N/A |
| Certainty of evidence | 22 | Present assessments of certainty (or confidence) in the body of evidence for each outcome assessed. | p.13-15, see eTable 4 in Supplement 1 |
| **DISCUSSION** | | |  |
| Discussion | 23a | Provide a general interpretation of the results in the context of other evidence. | p.15-18 |
|  | 23b | Discuss any limitations of the evidence included in the review. | p.18 |
|  | 23c | Discuss any limitations of the review processes used. | p.18 |
|  | 23d | Discuss implications of the results for practice, policy, and future research. | p.18 |
| **OTHER INFORMATION** | | |  |
| Registration and protocol | 24a | Provide registration information for the review, including register name and registration number, or state that the review was not registered. | p.6 |
|  | 24b | Indicate where the review protocol can be accessed, or state that a protocol was not prepared. | p.6 |
|  | 24c | Describe and explain any amendments to information provided at registration or in the protocol. | p.6 |
| Support | 25 | Describe sources of financial or non-financial support for the review, and the role of the funders or sponsors in the review. | p.3-4 |
| Competing interests | 26 | Declare any competing interests of review authors. | p.3 |
| Availability of data, code and other materials | 27 | Report which of the following are publicly available and where they can be found: template data collection forms; data extracted from included studies; data used for all analyses; analytic code; any other materials used in the review. | p. 4 |

*From:*  Page MJ, McKenzie JE, Bossuyt PM, Boutron I, Hoffmann TC, Mulrow CD, et al. The PRISMA 2020 statement: an updated guideline for reporting systematic reviews. BMJ 2021;372:n71. doi: 10.1136/bmj.n71

**eFigure 1.** Forest plot for meta-analysis of change in 6-minute walk test pre-procedure versus 4-6 weeks post-procedure in included studies


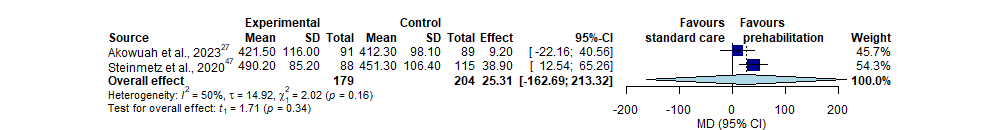


**eFigure 2.** Forest plot for meta-analysis of change in 6-minute walk test pre-procedure versus 12 weeks post-procedure in included studies


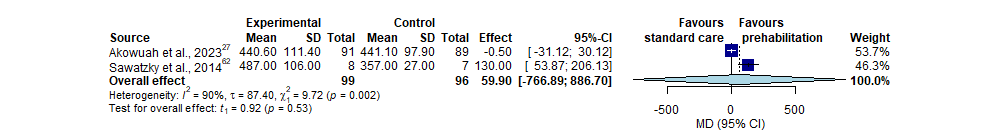


**eFigure 3.** Forest plot for meta-analysis of change in pre-procedure hand grip strength at baseline versus prior to the procedure in included studies


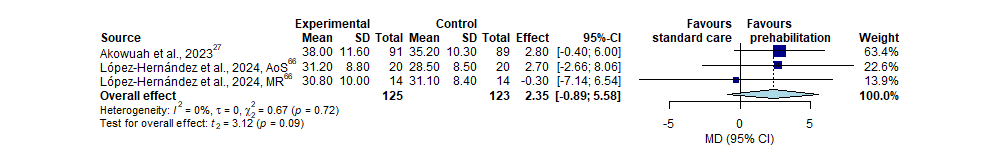


**eFigure 4.** Forest plot for meta-analysis of change in pre-procedure overall quality of life at baseline versus prior to the procedure in included studies


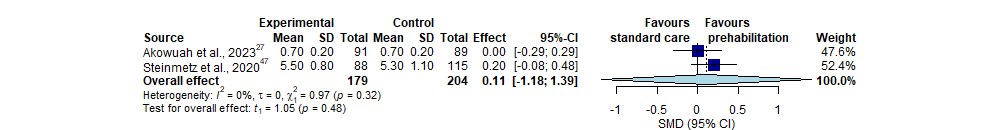


**eFigure 5.** Forest plot for meta-analysis of change in pre-procedure versus post-procedure overall quality of life in included studies


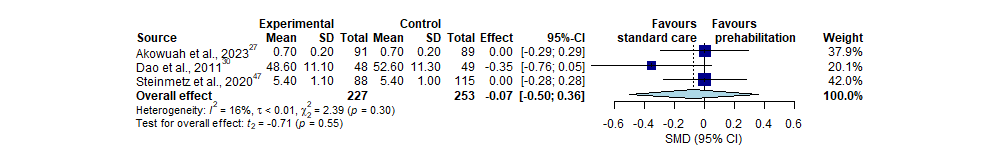


**eFigure 6.** Forest plot for meta-analysis of change in pre-procedure versus post-procedure anxiety in included studies


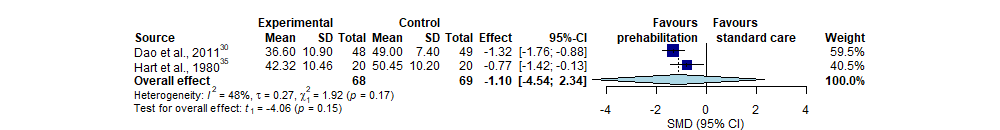


**eFigure 7.** Forest plot for meta-analysis of change in post-procedure delirium in included studies


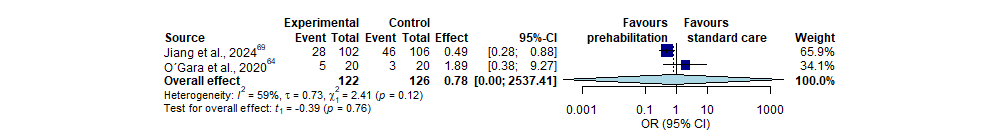


**eFigure 8.** Forest plot for meta-analysis of change in post-procedure infection in included studies


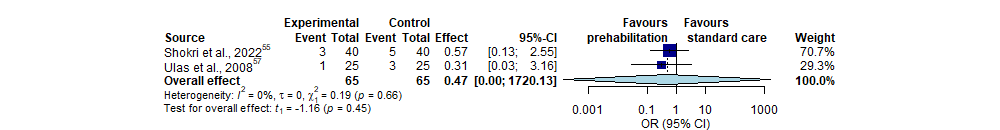


**eFigure 9.** Bubble plot showing the association between LOS and sex

**
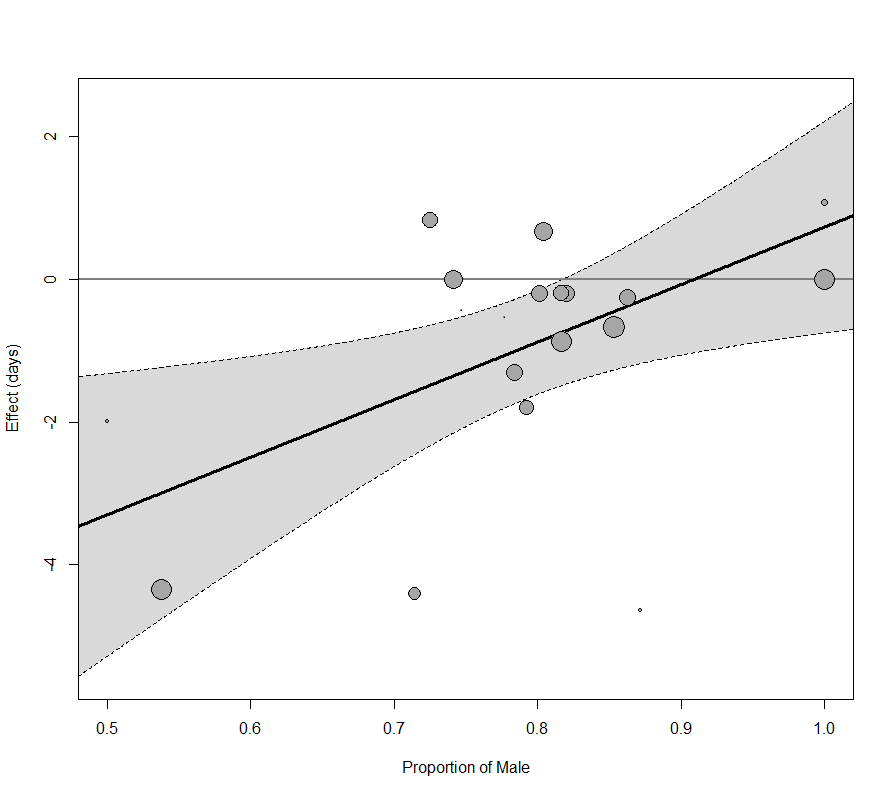
**

**eFigure 10**. Forest Plots of Recovery Status and Perioperative Functional Capacity Including Subgroup Analyses Uni- vs. Multimodal Prehab Programs.

**A** In-hospital length of stay (days)


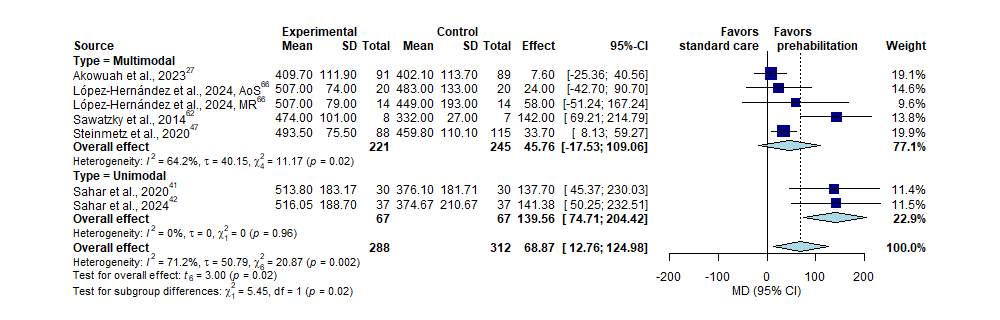

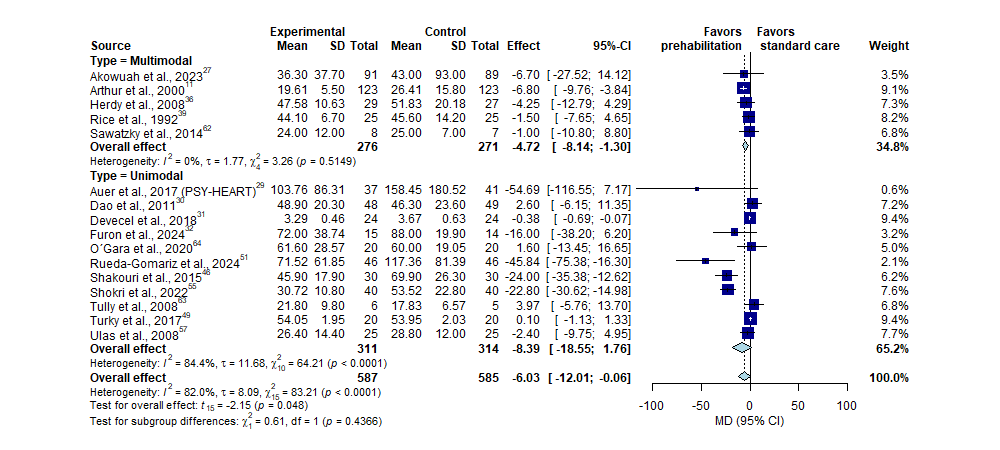

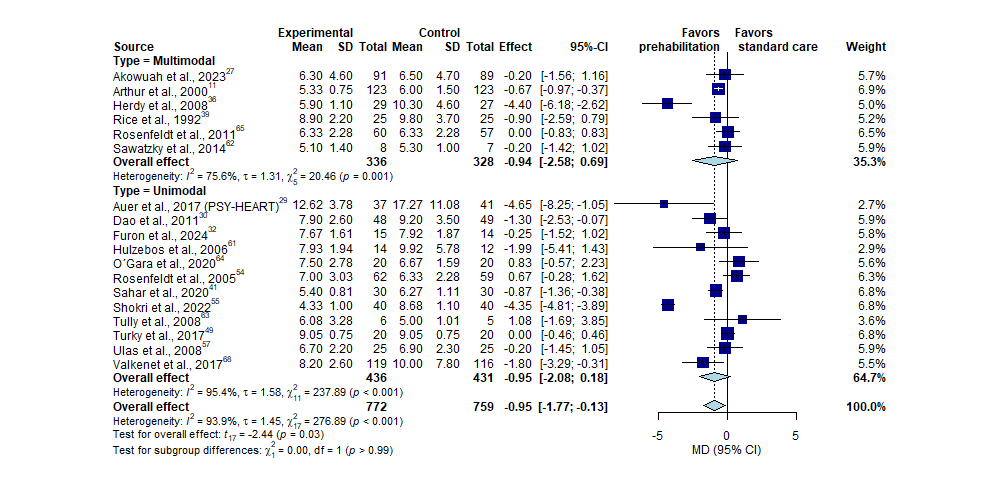


**C** 6-minute walk distance pre-procedure (meters)

**B** ICU length of stay (hours)


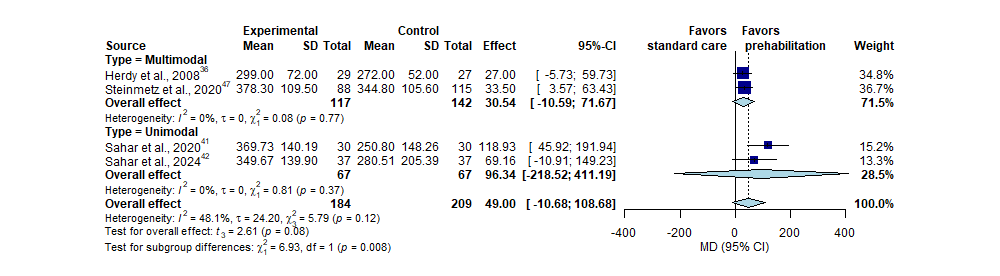


**D** 6-minute walk distance post-procedure (meters)

**eFigure 11**. Forest Plots of Selected Postoperative Complications Including Subgroup Analyses Uni- vs. Multimodal Prehab Programs.


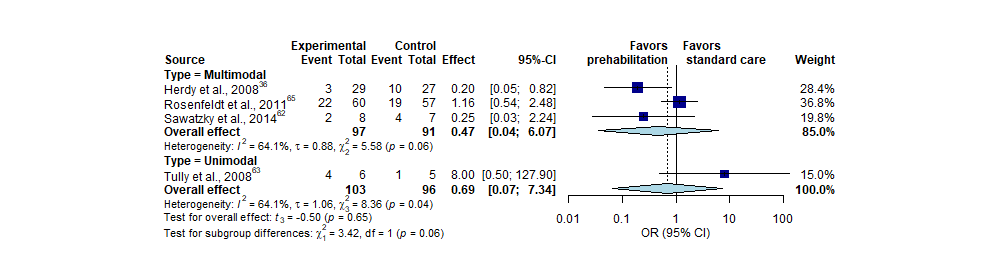


**B** Post-procedural pneumonia

**A** Post-procedural atrial fibrillation


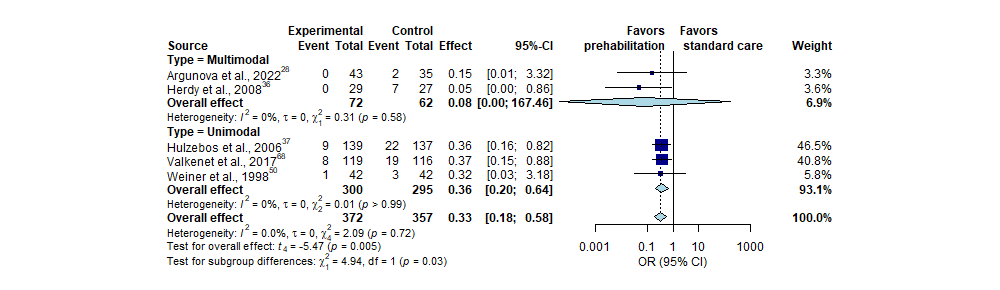


**C** Post-procedural atelectasis


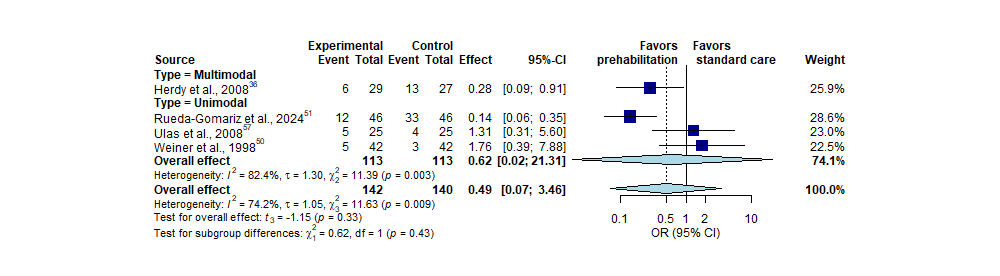

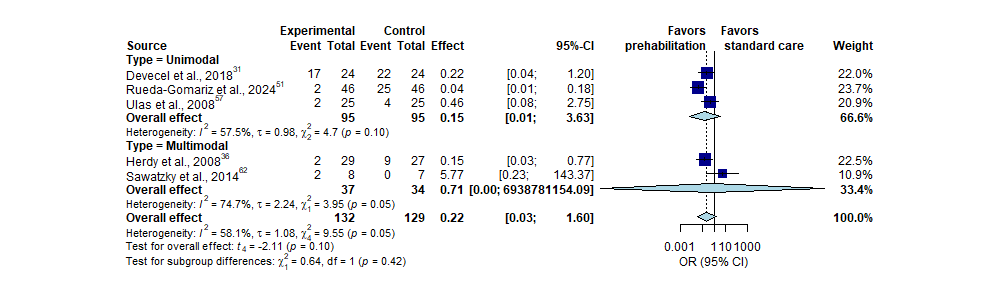


**D** Post-procedural pleural effusion


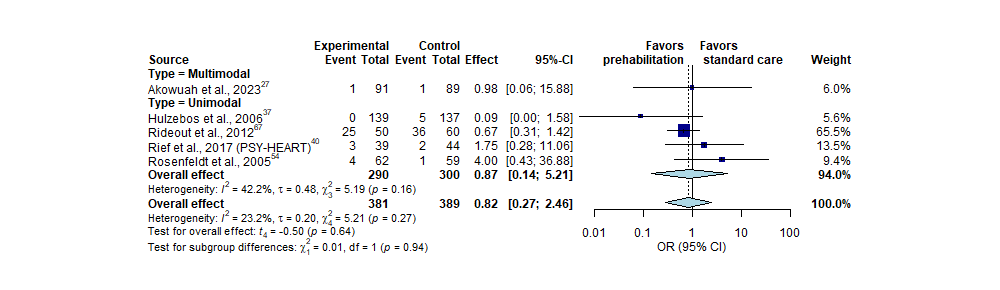


**E** All-cause mortality (follw up time including in- and out-hospital mortality)

**eFigure 12**. Forest Plots of In-hospital Length of Stay Including Subgroup Analyses According to Prehabduration at least 2 weeks vs. less than 2 weeks.


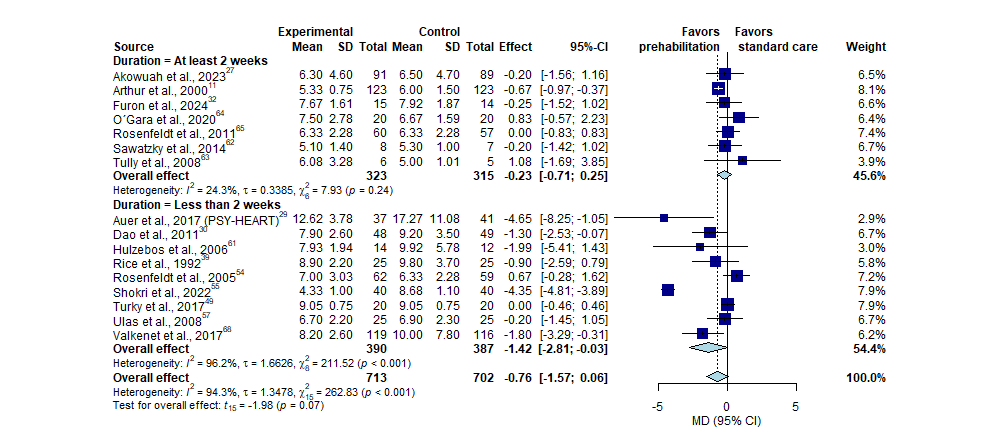


**A** In-hospital length of stay (days)


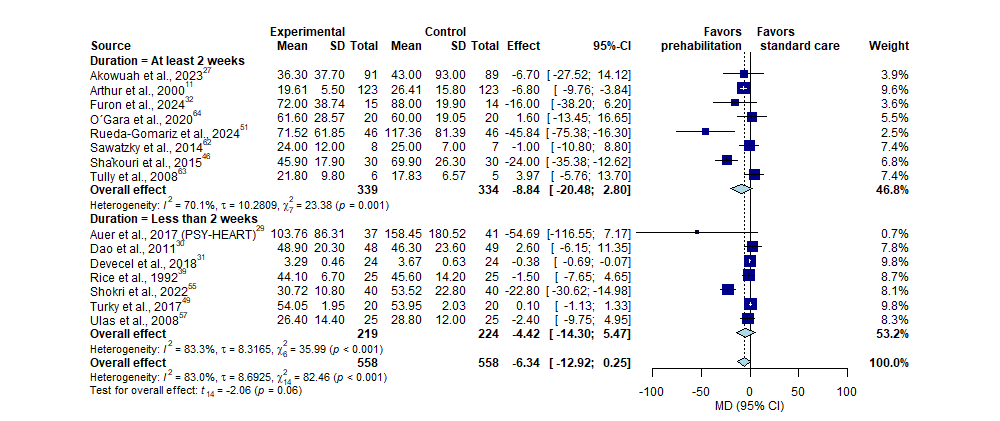


**B** ICU length of stay (hours)


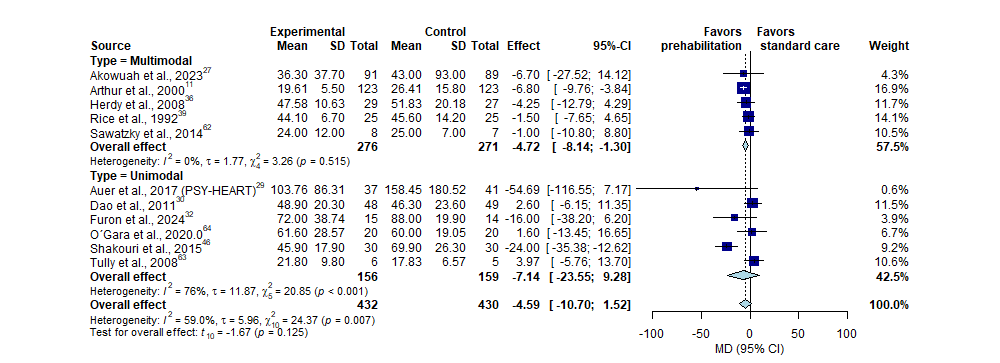

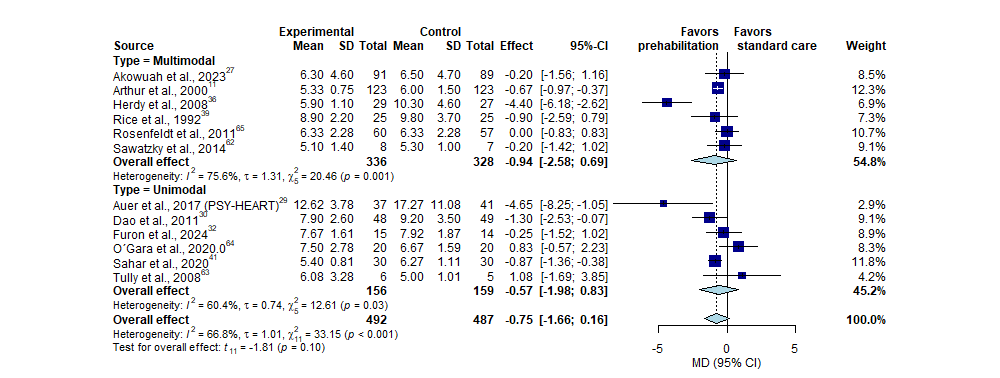
**eFigure 13.** Forest Plots of Recovery Status and Perioperative Functional Capacity Including Subgroup Analyses Uni- vs. Multimodal Prehab Programs without breathing only or supplement/medication only studies.

**B** ICU length of stay (hours)

**A** In-hospital length of stay (days)

**C** 6-minute walk distance pre-procedure (meters)


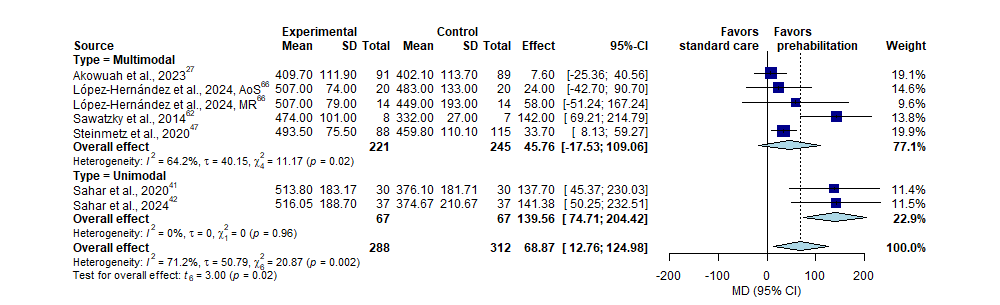


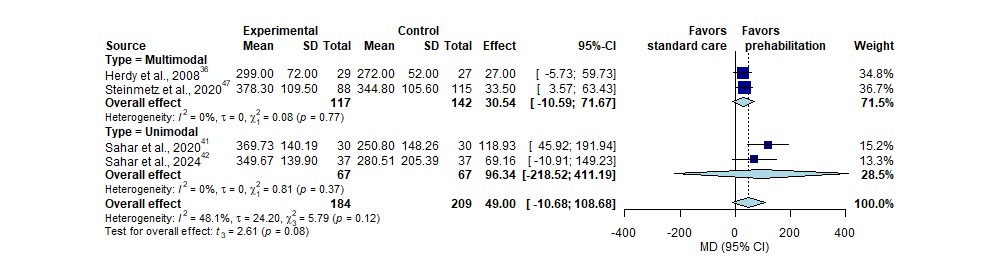


**D** 6-minute walk distance post-procedure (meters)


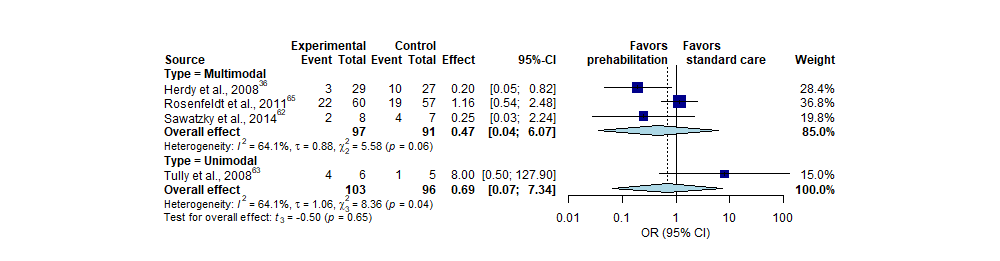
**eFigure 14.** Forest Plots of Selected Postoperative Complications Including Subgroup Analyses Uni- vs. Multimodal Prehab Programs without breathing only or supplement/medication only studies.

**A** Post-procedural atrial fibrillation

**B** Post-procedural pneumonia


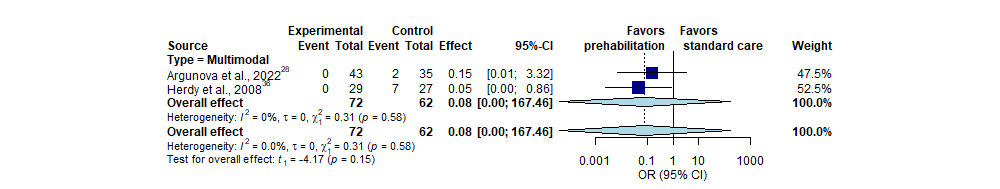


**C** Post-procedural atelectasis

**D** Post-procedural pleural effusion

**E** All-cause mortality (follw up time including in- and out-hospital mortality)


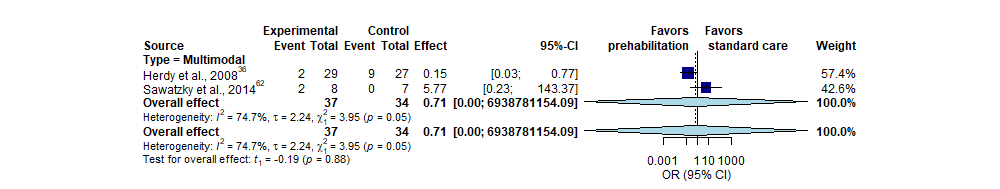


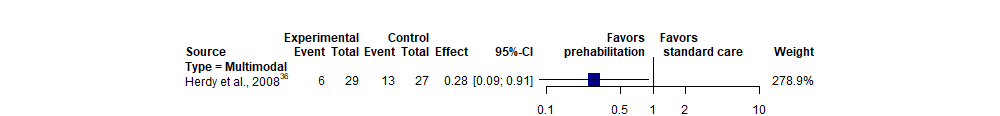


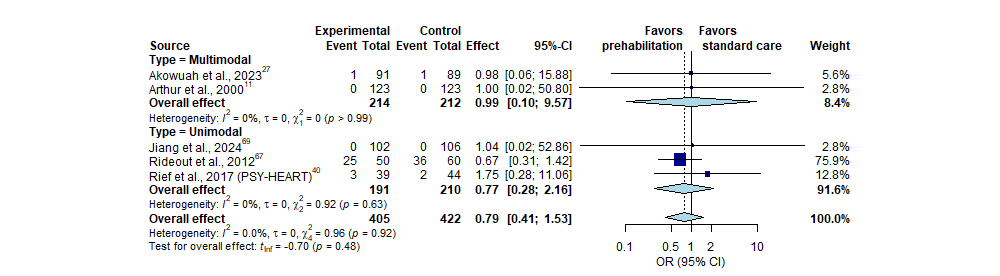


**eFigure 15.** Forest Plots of In-hospital Length of Stay Including Subgroup Analyses According to Prehabduration at least 2 weeks vs. less than 2 weeks without breathing only or supplement/medication only studies.


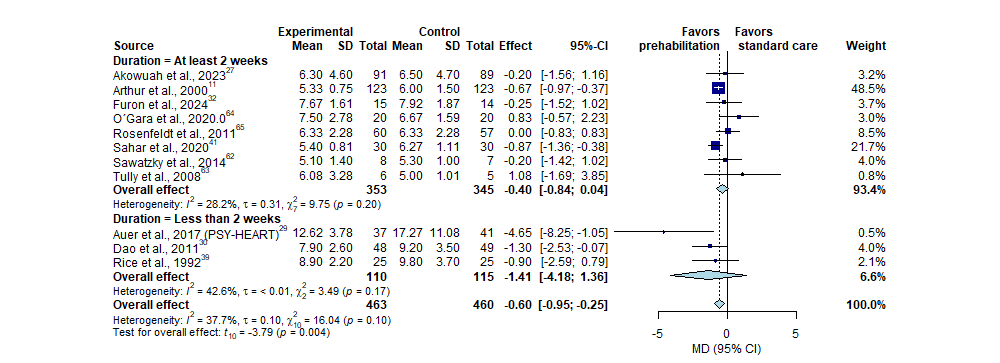


**A** In-hospital length of stay (days)

**B** ICU length of stay (hours)


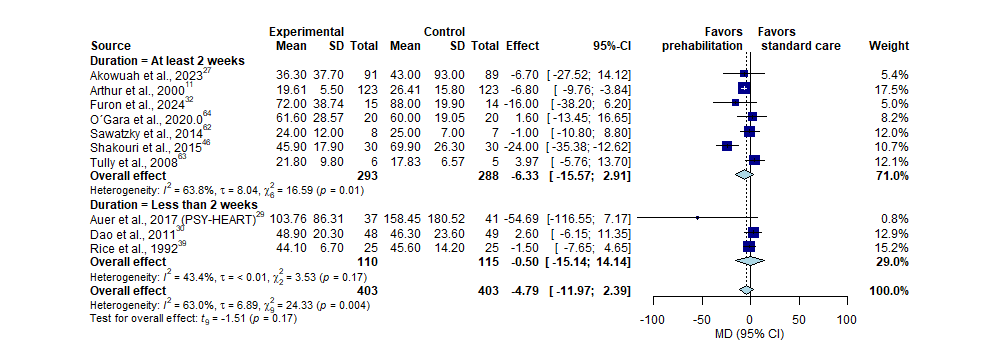

Supplement: Supplemental File 1 [file mmc1.docx]
